# Day-to-day fluctuations in motivation drive effort-based decision-making

**DOI:** 10.1073/pnas.2417964122

**Published:** 2025-03-17

**Authors:** Samuel R. C. Hewitt, Agnes Norbury, Quentin J. M. Huys, Tobias U. Hauser

**Affiliations:** ^a^Max Planck UCL Centre for Computational Psychiatry and Ageing Research, Queen Square Institute of Neurology, University College London, London WC1B 5EH, United Kingdom; ^b^Functional Imaging Laboratory, Department of Imaging Neuroscience, University College London, London WC1N 3AR, United Kingdom; ^c^Applied Computational Psychiatry Lab, Mental Health Neuroscience Department, Division of Psychiatry and Max Planck UCL Centre for Computational Psychiatry and Ageing Research, Queen Square Institute of Neurology, University College London, London W1T 7NF, United Kingdom; ^d^Department of Psychiatry and Psychotherapy, Faculty of Medicine, University Tuebingen, Tübingen 72076, Germany; ^e^German Center for Mental Health (DZPG), Project Site Tübingen, Tübingen 72076, Germany

**Keywords:** decision-making, reward, effort, motivation, longitudinal

## Abstract

Internal states like motivation fluctuate substantially over time. However, studies of the neurocomputational mechanims of motivated behavior have failed to capture this. Here, we examined how naturalistic ups and downs in state motivation influence the subjective value of reward and effort. In a microlongitudinal design (N = 155, state timepoints = 3,344, decision-making tasks = 845), we captured fluctuations in state and effort-based decision-making using smartphone-based momentary assessments as people went about their daily lives. We found that both state and trait have independent and multiplicative effects on decision-making. State–behavior coupling was particularly pronounced in individuals with higher trait apathy, meaning that their choices were even more state dependent. Using computational modeling, we demonstrate that state motivation prospectively boosted reward sensitivity, making people more willing to exert effort in future. Our results show that day-to-day fluctuations in state and cognition are tightly linked and critical for understanding fundamental human behaviors and mental ill-health.

## Significance

In the real-world, people make choices amid a complex array of fluctuating states. But little is known about how such fluctuations influence the computational processes which govern motivated behavior (e.g., effort-reward arbitration). Cross-sectional studies in cognitive neuroscience have assumed trait-like stability of neurocomputational mechanisms. Using densely repeated behavioral assessments, we show that normal day-to-day ups and downs in the motivation that people experience impact their choices by modulating momentary sensitivity to reward, a critical, underlying, computational mechanism. Motivation drives changes in the subjective value of reward both in the moment and in the future. These findings show that fundamental mechanisms of decision-making are subject to the rapid ebbs and flows of daily life.

Change is fundamental to human experiences. A prime example is feeling motivated, defined as a willingness to initiate and maintain goal-directed behaviors (1). Fluctuations in motivation appear to characterize day-to-day life (2). One day, you feel motivated to exercise or catch up with an old friend but the next day, this changes and you prefer to stay at home. Such rapid ebbs and flows in motivation are clearly dissociable from the slower, disabling fluctuations in psychiatric disorders like depression, where episodes of low motivation can persist for weeks to months (3, 4). While certain fluctuations are clearly tied to external events (e.g., expectations) or needs (e.g., hunger), many ups and downs seem to occur unpredictably and serendipitously.

Despite the widespread intuition that states like motivation change over time, quantitative experimental studies fall short in capturing this (5–13). In particular, quantitative, effort-reward arbitration tasks are commonly deployed at a single timepoint to study the neurocognitive mechanisms underlying motivation. These snapshot-like neurocognitive assessments indeed show (modest) associations with trait motivation (14) and motivation-related disorders such as depression (15–17), Schizophrenia (18, 19) or Parkinson’s disease (10, 20). However, the degree to which state fluctuations influence decision-making remains unclear. Computational modeling, which already identified the latent neurocognitive motivational processes that govern choices in these tasks (21–23), has the potential to yield a much richer understanding of human behavior by taking into account the complexity of naturalistic ebbs and flows in motivational state.

The case of motivation is not unique. Task-based studies in cognitive neuroscience have consistently ignored fluctuations over time. Variability in choices is usually attributed to error in the data collection, e.g., limited psychometric properties (24) or analysis, e.g., parameter estimation (25) or simple practice or boredom effects (26, 27). The prevailing assumption is that such tasks measure stable, trait-like functions and that state variability is not important in explaining choices or their underlying mechanisms (28). This is despite recent findings that very fast fluctuations (in the order of milliseconds to minutes) in subjective and brain states influence choices (29, 30).

To test the extent to which day-to-day state fluctuations drive choices, we conducted a longitudinal study and decomposed time-varying and time-invariant effects on behavior. Capturing state motivation and effort-based decision-making for 2 wk using smartphone-based assessments (mean datapoints per person = 26.4), we found that effort-reward arbitration is subject to both trait and state motivation. Effort-based choices were tightly coupled to the motivation people reported in that moment. Using a hierarchical computational model, we show that the subjective state drives choices by modulating current and future sensitivity to reward. This reveals that such tasks capture real-world, momentary experiences over and above trait-like characteristics. By disentangling their contributions with densely repeated measures, we can start to better understand how specific transient feelings and choices dynamically influence each other over time.

## Results

**Fig. 1.**
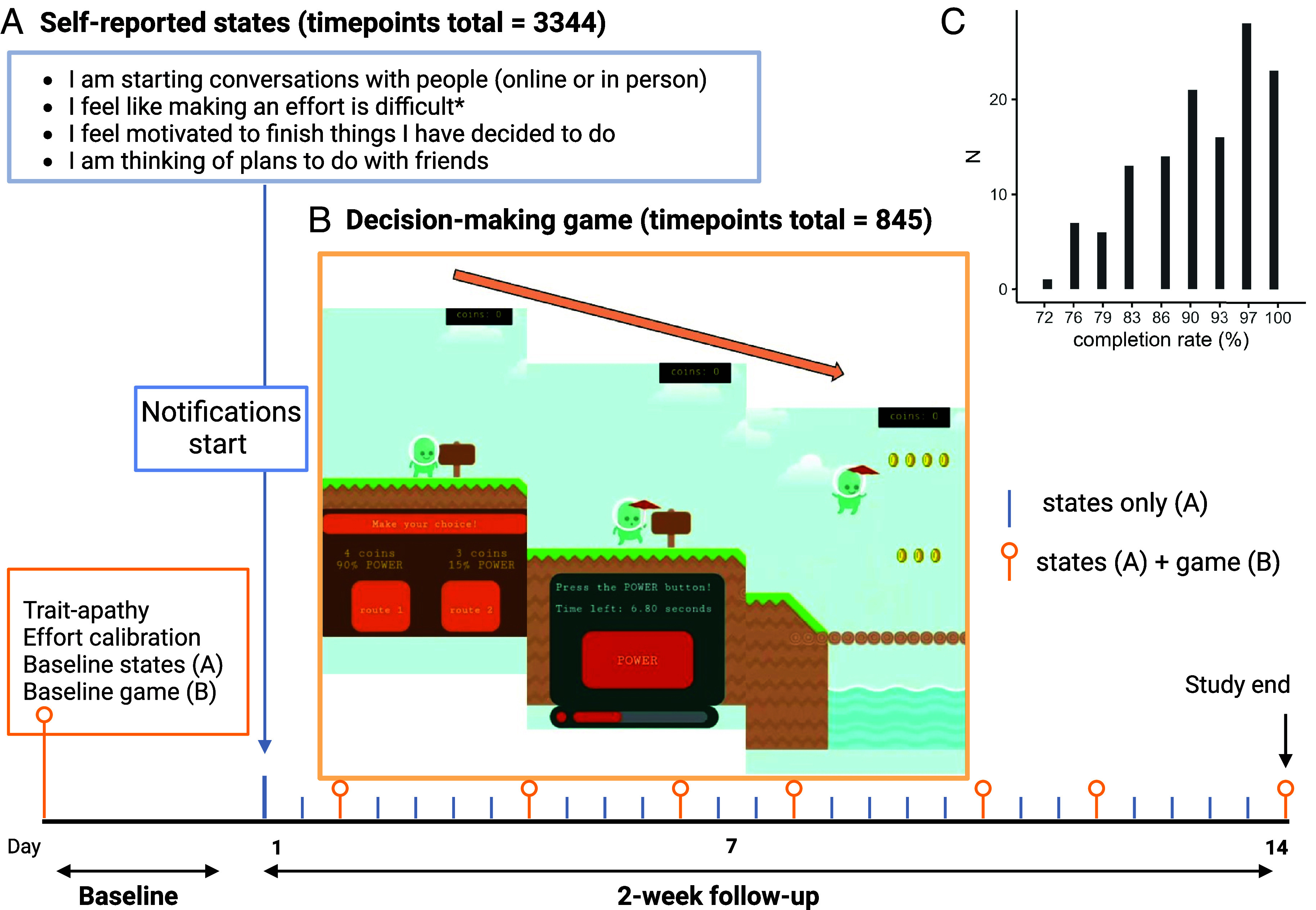
Microlongitudinal study design to determine the link between real-world, rapidly unfolding subjective states and decision-making. The study involved self-report of subjective state “right now” (*A*) and decision-making (*B*) over 15 d. This included a baseline session (day 0) with app enrollment, self-reported traits, maximum effort calibration, the first state and decision-making game. Follow-up began the next morning. (*A*) Participants received smartphone notifications twice per day (1 morning, 1 afternoon) to report subjective social and behavioral motivation right now (blue box, *reverse scored item), along with happiness, fatigue, and sleep quality (*Methods*). On alternate days (25% of study timepoints, orange marker), they also repeated the decision-making task and reported the state afterward. The order of task assessments was pseudorandomly counterbalanced between morning and afternoon (4 each). (*B*) Smartphone-based decision-making task. Trials begin with a choice between two routes varying in reward (coins) and effort (*Left*). Greater effort was manipulated as more button presses within 10 s (% of maximum). After making a choice, the participant presses POWER as fast as possible to fill the power bar within 10 s (*Middle*). When successful (success μ = 0.98), feedback is displayed and the character flies across the route to collect the coins associated with the chosen option (*Right*). (*C*) We required participants to provide valid data at ≥70% of the study notifications. From those included (N = 129), most participants (*y*-axis) provided valid data at most assessments (*x*-axis). Timepoints total = independent aggregate state and task measures available after exclusion criteria. Created with BioRender.com (31).

We examined the extent to which people naturally experience day-to-day fluctuations in motivation and how these impact their choices in a mircrolongitudinal study online with 155 adults (18 to 45 y, 50% female) from the general population. After trait motivation/apathy and baseline effort–reward arbitration assessments, participants reported subjective states and repeated cognitive tasks on their smartphones for 15 d (Fig. 1). After data cleaning, we analyzed data from 129 people [age = 32.4 ± 0.59 y (mean ± 1SEM), 52.7% female] who provided 3,344 momentary states [25.9 ± 0.2 (mean ± 1SEM), mode = 97% completion rate], and 845 decision-making games (6.6 ± 0.1, mode = 100% completion rate).

### Healthy People Experience Substantial Fluctuations in State Motivation.

**Fig. 2.**
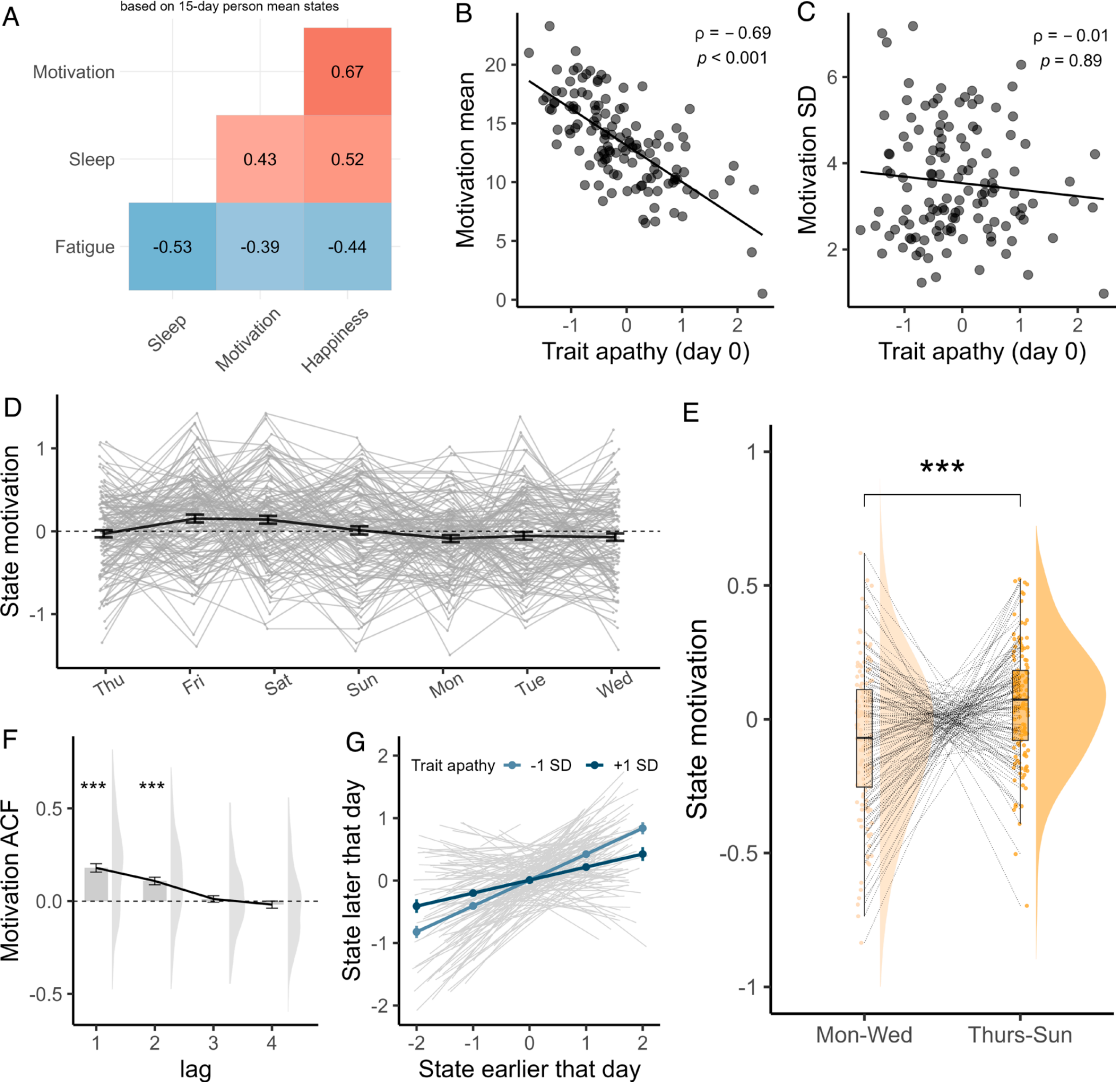
Naturalistic, day-to-day fluctuations in state. (*A*) Spearman rank correlations for mean states (over 15 d; all *P* < 0.001). People who reported greater motivation also reported greater happiness, better quality sleep, and lower fatigue. (*B*) Trait apathy (*x*-axis) was a strong negative predictor of 15-d mean (absolute) state motivation (*y*-axis). (*C*) Trait apathy (*x*-axis) was not related to the SD in state motivation (*y*-axis). (*D*) Person mean (normalized within-person) state motivation (gray) and group mean ± SE of mean (black) across weekdays (*x*-axis). Each gray line is an individual, which gives an idea of the substantial variability. The *x*-axis follows the study order, which began on a Thursday and *y*-axis is truncated to −1 and +1 SD from the mean to better visualise the group trajectory. (*E*) Mean (normalized within-person) state motivation for the first part of the week (Mon–Weds) compared with the second part (Thurs–Sunday) significantly differed, with greater motivation later in the week. (*F*) State motivation autocorrelation (*y*-axis, ACF = autocorrelation function) across participants indicating the mean correlation between state fluctuations at a given time and previous timepoints up to lag-4 (bar = group mean, errors = group mean ± SEM). (*G*) Autocorrelation between state motivation earlier in the day (first notification between 09:00 and 13:00, *x*-axis) compared with state later on the same day (14:00 and 18:00, *y*-axis) was typically strong and positive (gray slopes are individuals). The negative moderation effect of trait apathy on the autocorrelation of state fluctuations is overlaid as fitted mean ± SEM at each level state, for trait apathy at −1 and +1 SD from the mean. People with greater apathy (dark blue) experienced a weaker, positive relationship between states within a day meaning that states early in the day were not as strongly sustained to the afternoon. ***paired *t* test *P* < 0.001.

First, we investigated whether and how subjective state motivation fluctuates over time (Fig. 1). Within-people (over time), state motivation was significantly positively correlated with happiness (repeated measures *r*_(3214)_ = 0.51, 95% CI [0.48, 0.54], *P* < 0.001) and sleep quality (the previous night, repeated measures *r*_(2031)_ = 0.24, 95% CI [0.19, 0.28], *P* < 0.001) and anticorrelated with fatigue (repeated measures *r*_(3214)_ = −0.43, 95% CI [−0.47, −0.4], *P* < 0.001). We observed similar patterns for between-person correlations (based on 15-d averages). People who, on average, reported greater motivation also reported better sleep (*rho*_(127)_ = 0.43, *P* < 0.001), less fatigue (*rho*_(127)_ = −0.39, *P* < 0.001), and greater happiness (*rho*_(127)_ = 0.67, *P* < 0.001; Fig. 2*A*), which replicates the trait literature (14, 32).

Trait apathy (factor analysis derived) at baseline was a significant negative predictor of mean state motivation (β = −3.13, 95% CI [−3.71, −2.56], *P* < 0.001, *R*^2^ = 0.49; Fig. 2*B*) but not its SD (β = −0.14, 95% CI [−0.41, 0.13], *P* = 0.31; *R*^2^ = 0.01; Fig. 2*C*). This confirms the construct validity of our state measure, since reduced average motivation is the defining feature of trait apathy.

State motivation fluctuated systematically across the week. People experienced significant changes across weekdays (*F*(6,985) = 3.4, *P* = 0.002, η^2^ = 0.01; Fig. 2*D*) with increases in their state motivation toward the end of the week (Thursday–Sunday) compared with the first part (Monday–Wednesday) (paired *t*_(128)_ = −3.19, *P* = 0.002, mean difference = −0.13, 95% CI [−0.22, −0.05]; *d* = 0.28, Fig. 2*E*), which was driven by both behavioral and social motivation (*SI Appendix*). This reveals the ecological validity of our state measure, reflecting popular intuitions about day-to-day fluctuations in this state (2).

Trait apathy also predicted how state motivation unfolded over time. To test this, we estimated the autocorrelation of state motivation (temporal relatedness or inertia) which was positive and significant within a day (β_state-1_ = 0.31, 95% CI [0.24, 0.37], *P* < 0.001, normalized within-person), meaning that feeling motivated in the morning was typically maintained to later that day. The temporal association then decreased with increasing time distance (Fig. 2*F*), similar to other affective states (33). Interestingly, trait apathy predicted reduced state autocorrelation within the day (β_state-1*trait-apathy_ = −0.11, 95% CI [−0.19, −0.04], *P* = 0.004; Fig. 2*G*). This reveals that trait apathy predicted motivation which was both lower (on average) and less strongly sustained from the morning to the afternoon.

### Fluctuations in Decision-Making Using Ecological Momentary Assessments.

The previous analyses confirmed that state motivation fluctuates meaningfully over time. Our goal was to test whether such fluctuations are related to effort-based decision-making. We developed a smartphone-compatible, gamified, reward–effort discounting task (median completion time = 6.5 min), similar to previous versions (15, 34). Briefly, the task involves crossings (trials) where participants choose between two routes (Fig. 1*B*). One route is associated with greater reward (converted to monetary bonus) and greater physical effort (% of participant maximum calibrated at baseline) in a two-alternative forced-choice format. Participants select their preferred route (no time limit) and exert effort by pressing a power button as fast as possible to fill the power bar (10 s).

**Fig. 3.**
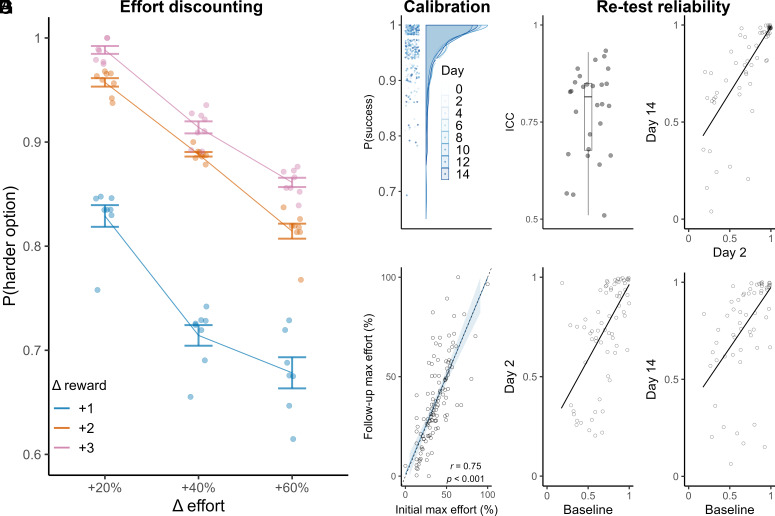
Reliability and validity of the smartphone assessment of effort-based decision-making. In this plot, filled circles are days and empty circles are participants. (*A*) Probability of choosing the harder option (*y*-axis) depended on the difference in the reward value (coloured errorbar = group mean ± SEM, points = game (day) group mean ± SEM), the difference in the effort-values (*x*-axis) and their interaction. This pattern is consistent with linear reward–effort discounting. (*B*) The mean probability of successfully receiving coins (*y*-axis) for each game was consistently near to 1. (*C*) Maximum effort was standardized to each person through a calibration procedure at baseline (day 0; *x*-axis, normalized %). The initial maximum effort (*x*-axis) calibrated at the start of the first game was a strong predictor of the mean 100% effort across the remainder of the study (days 2 to 14, *y*-axis, normalized %). The ordinary least squared regression (x ~ y) ± SE (blue) overlaps with an exact fit (dashed-line, intercept = 0, slope = 1) indicating excellent calibration of effort-level across the study. For full details of the calibration procedure, see *Methods* and *SI Appendix*. (*D*) Test–retest reliability (ICC) of mean probability of choosing the harder option for each of 28 possible 2-game combinations. (*E*–*G*) The reliability of *P*{harder option} between example game combinations: Baseline (Day 0), Day 2 and Day 14.

The game elicited reward–effort discounting similar to traditional laboratory tasks (Fig. 3*A*). People were more likely to choose the harder option when the difference in effort was less pronounced (*F*(2, 56) = 532,9, *P* < 0.001, η^2^ = 0.37) and when the potential reward was greater (difference high-effort reward—low-effort reward; *F*(2, 56) = 851.1, *P* < 0.001, η^2^ = 0.58). The probability of choosing the harder effort option also varied as a function of the interaction between the difference in effort and the difference in reward [*F*(4, 56) = 19.5, *P* < 0.001; η^2^ = 0.03], meaning that choices were based on the combined value of the reward and effort consistent with reward–effort discounting (Fig. 3*A*). The mean probability of success on each trial (i.e., filling the power bar and receiving reward) was near to 1 (μ ± SEM = 0.98 ± 0.01) and did not significantly differ across the 8 games in the study (binomial β_day_ = −0.01 95% CI [−0.04, 0.05], *P* = 0.95; Fig. 3*B*). This confirms that choices were based on reward–effort (not probability) discounting.

To confirm that the task was comparable across timepoints, we analyzed participants overall behavior. The maximum effort participants could achieve at baseline was well calibrated because it strongly predicted their maximum across all subsequent days (*r* = 0.75, *P* < 0.001 95% CI [0.67, 0.82]; Fig. 3*C*). The test–retest reliability of physical effort (mean lag between power button presses) and the probability of choosing the harder option (*P*{harder option}, Fig. 3*A*) were both moderate-high across all (total = 28) 2-game combinations (mean ICC_P(harder option)_ = 0.78 ± 0.02, 95% CI [0.74, 0.82]; mean ICC_effort_ = 0.85 ± 0.01, 95% CI [0.83, 0.87]; Fig. 3*D*). This shows that our brief smartphone game robustly captured variability in effort-based decision-making with minimal impact on participant’s daily life. Next, we tested whether states were related to choice fluctuations.

### Trait Apathy Predicts Reduced Willingness to Exert Effort for Reward.

**Fig. 4.**
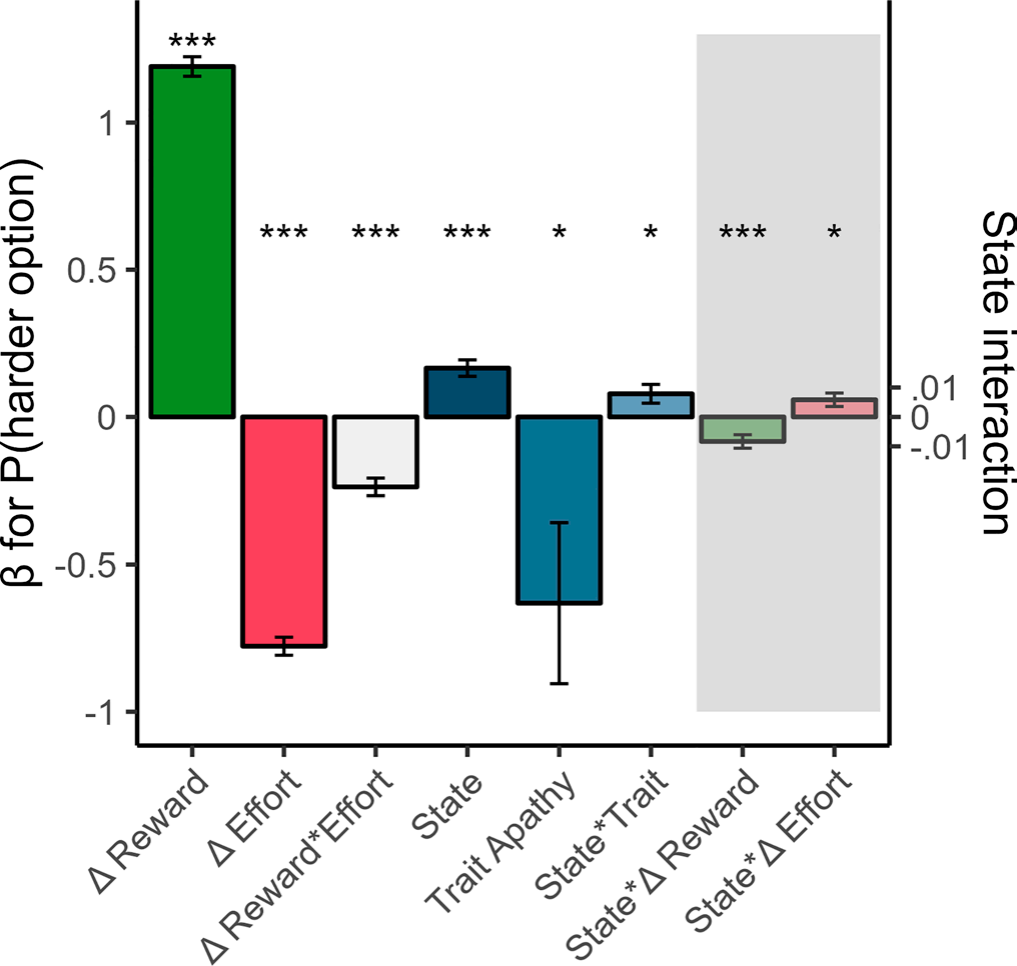
Fixed effects for predictors of p(harder option) on each trial in the study. (Gray area = state-reward and state-effort interaction effects estimated independently) *P < 0.05, **P < 0.01 ***P < 0.001. Δ = difference on a given trial.

First, we confirmed that the smartphone game replicated known links between effort-based choices and trait apathy (6, 13, 14, 35). We summarized effort-based choices on a given day as the mean difference in effort between the chosen and unchosen options (%). This measure captures willingness to exert effort for reward at that time (amalgamates preference for reward and effort into a single choice measure). As expected, trait apathy predicted a reduction in willingness to exert effort over the next 2-wk (β_trait-apathy_ = −4.13%, 95% CI [−7.78, −0.47], *P* = 0.028; Fig. 4*A*). This confirms that the smartphone game had good external validity and extends previous work to show that trait apathy negatively predicts future effort-based choices.

### State Fluctuations Are Coupled with Greater Willingness to Exert Effort for Reward.

If the game captures how motivated people feel in that very moment, then choices it elicited should be positively associated with fluctuations in state motivation (independent of between-person characteristics). Indeed, we found that on days when people felt more motivated, they chose to make more effort for reward (β_State_ = 1.39, 95% CI [0.53, 2.26], *P* = 0.002; Fig. 4*B*, normalized within-person). This was entirely independent of trait apathy (correlation of fixed effects: β_State ~_ β_trait-apathy_ = 0.01) or other covariates (age = 0.14, 95% CI [−2.97, 3.24], *P* = 0.93; sex(male) = −0.62, 95% CI [−6.85, 5.6], *P* = 0.84). Given that participants played the tasks up to 8 times, we also assessed whether practice effects contributed to task performance (27). There were modest practice effects on decision-making as defined by a plateauing power-law curve of greater willingness to exert effort for reward over time (mean *R*^2^ across participants = 0.34, 95% CI [0.29, 0.39]). However, the state effect explained significantly more variance in effort-based choices than practice alone (paired *t*_(115)_ = 8.14, *P* < 0.001, mean difference in *R*^2^ = 0.13, 95% CI [0.1, 0.16]; *SI Appendix*).

Next, we confirmed whether the state effect was specific to motivation (given substantial state-correlations, Fig. 2). To do this, we tested whether the other states (happiness, fatigue, and sleep quality) were associated with choices and whether this association held while controlling for state motivation within the same model. Choices on a given day were not related to state happiness (β_happiness_ = −0.15, 95% CI [−0.89, 0.59], *P* = 0.69), and a model with state motivation outperformed happiness alone in a model comparison (likelihood ratio test (X^2^_(1)_ = 12.8, *P* < 0.001). Critically, we also confirmed the superiority of state motivation when comparing it with the effect of self-reported fatigue and sleep quality (Fig. 4*C* and *SI Appendix*). In summary, we observed a highly specific effect, where only state motivation had a significant, positive association with willingness to exert effort.

### Trait Apathy Strengthens the Link between States and Choices.

Based on theories that apathy modulates the likelihood of engaging in goal-directed behavior (23), we investigated whether trait motivation modulated the state effect. We found a significant, positive state–trait interaction effect on effort-based choices (β_State*trait-apathy_ = 1.19, 95% CI [0.14, 2.24], *P* = 0.023; Fig. 4*D*). This means that more apathetic participants showed a stronger coupling between how motivated they felt in the moment and how willing they were to exert effort for reward. This sheds light on the effect of trait apathy, showing that it modulates the extent to which current feelings predict choices, making trait apathy particularly debilitating when in a low motivation state.

We also confirmed the specificity of the trait effect by estimating the effect of trait depression–anxiety and trait impulsivity which are both associated with trait apathy (36, 37) and behavioral variability (33, 38). This revealed that neither depression–anxiety nor impulsivity were related to choices or the coupling between states and choices (*SI Appendix*).

State motivation was not only associated with choices but also response vigor (speed of button pressing). In general, state motivation predicted increased response vigor (β = 1.04, 95% CI [0.69, 1.39], *P* < 0.001) but trait apathy was unrelated (β = −0.7,95% CI [−5.81, 4.4], *P* = 0.79). As with decision-making itself, the effect of state motivation on response vigor was even greater in people with higher trait apathy (β = 0.52, 95% CI [0.09, 0.95], *P* = 0.017). This shows that the state–trait interaction extended from cognition to action.

**Fig. 5.**
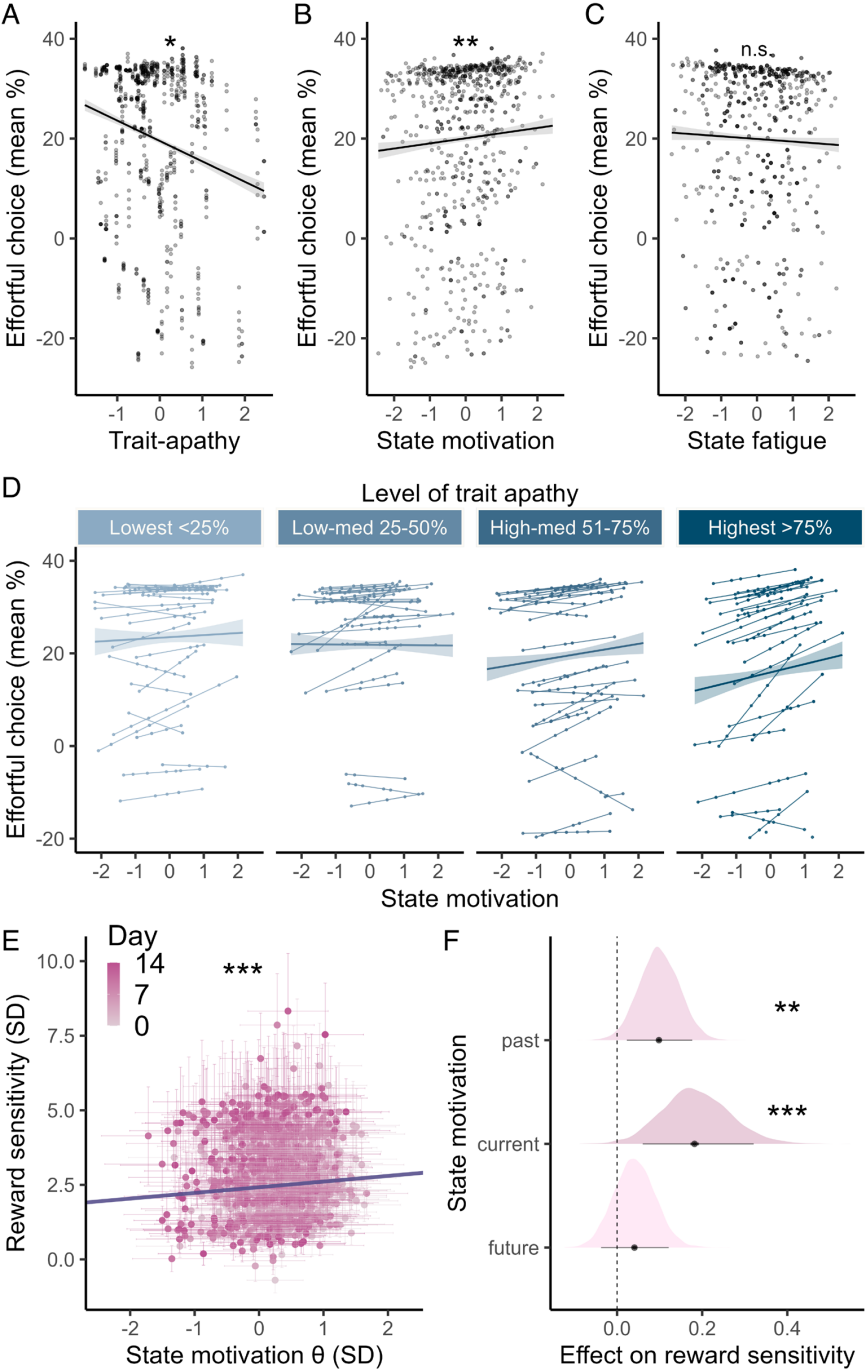
State motivation modulated effort-based decision-making by increasing reward sensitivity. Each point represents one game. (*A*) Main effect of trait apathy (*x*-axis) on choices [*y*-axis = conditional fitted mean (% effort chosen–unchosen)]. (*B*) Main effect of state motivation relative to 15-d person mean (*x*-axis) on choices (*y*-axis = conditional fitted mean % effort chosen—unchosen for each game). (*C*) The relationship between state fatigue (*x*-axis) and choices was not significant after controlling for state motivation (also the case for state happiness and sleep quality, *SI Appendix*). (*D*) State–trait interaction effect on behavior (slope increases at greater levels of apathy). In (*A*–*D*), the fixed effect (marginal predicted value) ± residuals are plotted as the smoothed regression line. (*E*) The latent motivational state (θ, *x*-axis) was positively associated with the latent decision-making parameter, reward sensitivity on a given day (*y*-axis). Errors = posterior mean ± SD. Purple solid line = posterior mean coefficient for the effect of current state motivation (β_stateR_ = 0.19, Fig. 4*F*, *Middle* row), with intercept set arbitrarily for visualization as mean (reward sensitivity). (*F*) Posterior mean coefficients for the effect of state motivation on reward sensitivity at different points in time. The past and current states were associated with greater reward sensitivity on a given day (*Top*, *Middle* row), but the future state (*Bottom* row) was not. Note that the current state effect was modeled first, independently. Post hoc, the past and future states were compared directly within a subsequent model and both assigned the empirical prior mean and SD of the current state effect. This explains the increased certainty (narrower distribution) of the posterior for the effects of past and future states. (*A*–*C*) *P < 0.05, **P < 0.01; (*E* and *F*) **P(Direction) ≥ 0.97, ***P(Direction) ≥ 0.99.

Using a logistic mixed effects model, we also confirmed that the effects of state motivation (β = 0.17, 95% CI [0.11, 0.22], *P* < 0.001), trait apathy (β = −0.63, 95% CI [−1.17, −0.1], *P* = 0.02), and their interaction (β = 0.08, 95% CI [0.02, 0.14], *P* = 0.01) were robust predictors of the probability of choosing the harder option on each trial while controlling for the large impact of the difference in reward offered (β = 1.19, 95% CI [1.13, 1.26], *P* < 0.001), difference in effort required (β = −0.78 ,95% CI [−0.84, −0.72], *P* < 0.001), and their interaction (β = −0.24, 95% CI [−0.3, −0.18], *P* < 0.001; Fig. 5). This confirms that the state effect was robust over and above the task options. Using a separate model to test the state modulation of choices as a function of reward and effort, we found that state motivation increased p(harder option) by significantly modulating the difference in reward (β = −0.08, 95% CI [−0.13, −0.04], *P* < 0.001) and, to a lesser extent, the difference in effort (β = −0.06, 95% CI [0.01, 0.1], *P* = 0.01, Fig. 5). Post hoc comparisons at each level of reward revealed that the state effect was strongest when the difference in reward was smallest, suggesting that the state was associated with a greater sensitivity to small differences in reward (*SI Appendix*). We next formally investigated how state motivation influenced the subjective value of reward and effort with a hierarchical generative model.

### State–Choice Coupling Is Driven by Fluctuating Reward Sensitivities.

We next investigated *how* the fluctuations in motivational states were associated with choices using computational modeling. Previous studies showed that individual differences in sensitivity to reward and effort govern the likelihood of engaging in effortful behavior (1), meaning that either fluctuations in reward sensitivity (rewards are more valuable) or effort sensitivity (effort is more costly) can explain changes in choices. Building on our previous work (34), we developed a hierarchical, generative model to test whether the coupling between states and choices was driven more by fluctuations in sensitivity to the reward offered or the effort required. We allowed a (person-centered) latent motivational state *θ* (derived from a hierarchical item response theory model) to influence the subjective value of each choice by modulating both reward and effort sensitivity on that day. To determine the state effects, we confirmed whether the 90% credible intervals excluded zero and also report the probability of direction (P(Direction)) which represents the likelihood of the existence of the effect (39).

This computational modeling analysis revealed that motivational state *θ* was significantly positively associated with reward sensitivity (β_state-rewSens_ = 0.19 ± 0.08 (SD), 90% CI [0.06, 0.32], *P*(Direction) = 0.99; Fig. 4 *E* and *F*) but not effort sensitivity (β_state-effSens_ = 0.29 ± 0.32 (SD), 90% CI [−0.23, 0.81], *P*(Direction) = 0.82). This means that state motivation had a specific effect on reward but not effort perception. Rewards were subjectively more valuable when people felt more motivated which shifted their choices to harder options.

### State Motivation Precedes Changes in Reward Sensitivity.

The previous analyses revealed that choices were coupled with state motivation because of changes in reward sensitivity but could not address the directionality of this relationship. In the absence of a causal manipulation, we approached this by looking at the temporal relationship between state and reward sensitivity fluctuations, assuming that temporal association is necessary for causation (40). We tested whether reward sensitivity was more related to the past motivation (suggesting the latent state drives reward perception) or the next (future) motivation (suggesting reward perception drives the latent state). Directly comparing these effects (within the same model) revealed that the reward sensitivity at a given moment was positively associated with the past motivational state (β_state-1_ = 0.1 ± 0.05 (SD), 90% CI [0.02, 0.17], *P*(Direction) = 0.98, Fig. 5*F*) but the same reward sensitivity was not associated with the future motivational state (β_state+1_ = 0.04 ± 0.05 (SD), 90% CI [−0.04, 0.12], *P*(Direction) = 0.8, Fig. 4*F*). Moreover, the impact of the preceding and concurrent state motivation did not meaningfully differ from each other ([β_state_ − β_state-1_] = 0.09 ± 0.09 (SD), 90% CI [−0.07, 0.24], *P*(Direction) = 0.82). This shows that reward sensitivity was coupled with state motivation not only at the time of choice but also before (and not afterward). This suggests that state motivation directly drives future effort-based choices by modulating the subjective value of reward but not vice versa.

## Discussion

In a large intensive longitudinal study, we found that effort-based choices are subject to day-to-day fluctuations in state motivation. When feeling more motivated than normal, the subjective value of reward increased and shifted preferences to exert more effort. Critically, state fluctuations drove fluctuations in future sensitivity to reward.

We designed a user-friendly study with brief, reliable, and densely repeated assessments. This allowed tracking of decision-making mechanisms with a high temporal resolution during day-to-day life and identified directional state effects, confirming that traditional cross-sectional studies fail to capture important variability. Our results speak directly to the ongoing discussion regarding limited reliability of neurocognitive assessments (41, 42), highlighting that state motivation is an important source of behavioral variance. Together, our results show that behavioral assessments, such as reward-effort decision-making, are sensitive to the rapid ebbs and flows of subjective experience (over and above trait-like characteristics), making them great assets for understanding real-world states.

State motivation influenced choices by increasing reward sensitivity. This means that when feeling more motivated, people weigh rewards more strongly than the effort accompanying these choices. We also found that choices were coupled with state motivation but not other feelings (e.g., happiness), even though these were correlated both within and between people. This is important because reward sensitivity is often blunted in conditions where low motivation persists over time, such as depression (43, 44). Here, we show that this reward sensitivity effect goes beyond mental illness and is evident even in typical, day-to-day changes in motivation. Our data also suggest that it is low motivation (rather than happiness) that drives reduced reward sensitivity in depression, which future studies could test directly.

Besides depression, apathy (i.e., lack of trait motivation) is a hallmark symptom of many neuropsychiatric disorders (23). We found that trait apathy predicted reduced state motivation level, reduced state inertia (i.e., increased state fluctuation, but no change in variance) and a stronger coupling between state motivation and choices. This state–trait interaction (which impacted the states themselves, decision-making, and response vigor) may be due to differences in motivation resources and their allocation. We propose that motivation is a limited resource with stable-trait and fluctuating-state components. When the stable component is high (low trait apathy), people rely less on how they feel in the moment and draw on greater motivational resources in reserve, allowing them to keep going even when they do not feel like it. For people who experience low stable component (high trait apathy), the state has a greater weight on motivation, leading to stronger state–behavior coupling. There are several speculative explanations for greater state weight, including differences in self-efficacy or beliefs about motivation, interoception (e.g., sensitivity to physiological changes), and compensation (e.g., working harder today to make up for yesterday). The mechanisms for differences in the impact of states are an interesting avenue for future intensive longitudinal studies.

Our finding that the state is primarily acting on reward sensitivity expands prior work suggesting that trait apathy is generally associated with effort sensitivity (35). In our data, the relationship between trait apathy and effort sensitivity was positive but did not reach significance, likely because our sample did not include clinical apathy and therefore, the range of trait apathy was limited. Future studies could investigate whether a state-driven reward sensitivity transfers to a trait-like effort sensitivity when apathy becomes more trait-like in clinical cohorts. Future studies might also actively or passively collect data on real-world activity, circadian rhythm, or chronotype (see, e.g., ref. 45) to determine how these predict state-changes in decision-making.

In this task, we measured effort as button presses because of the smartphone-based assessment (similar to, e.g., refs. 34 and 46) and found that the effort substantially impacted choices. This is reassuring that the calibrated effort was a meaningful cost. However, this may not have been as physically aversive as other methods (e.g., force grippers), or in tasks without gamification. It is possible that state motivation more strongly affects effort sensitivity when effort requirements are greater in the real-world (e.g., running a long distance).

The neural mechanisms associated with motivational state fluctuations and state-choice coupling remain unknown. In effort-based tasks (similar to ours), activity in human ventral striatum varies positively with prospective reward and the anterior cingulate cortex plays a key role in effort-reward arbitration (23, 47–49) Future neuroimaging studies could reveal whether activity in such circuits also predicts state-choice fluctuations, similar to the brain-behavior coupling seen on faster, minute-to-minute timescales (29, 50) and potentially differentiate people who persist in low motivation–reward insensitive states.

We hope this work inspires a transition from lab-based, cross-sectional studies to dense longitudinal designs, where computational mechanisms unfold over time and predict future behavior. Embedding our studies within the ebb and flow of daily life has the potential to reveal many new insights about the complex interplay between human affect, cognition, and behavior.

## Methods

### Participants.

The study was approved by the University College London Research Ethics Committee (reference: 15301/001) and all participants provided informed consent. Participants were 155 healthy adults (50% female) recruited online (https://prolific.co/), residing in the United Kingdom. Inclusion criteria were aged 18 to 45 y, fluency in English and smartphone ownership. Exclusion criteria were history of neurological, psychiatric or other chronic health condition, medication use for any reason, and undergoing stressful life events (e.g., bereavement, divorce, financial hardship).

### Design and Procedure.

The study had two stages: baseline and follow-up (Fig. 1). All study assessments were conducted on participant’s own smartphones within the Brain Explorer Android or iOS app (www.brainexplorer.net). At baseline, participants provided demographic information and completed trait questionnaires. They reported their initial subjective state and completed the first decision-making task (practice, calibration and game 1).

Follow-up began from the following morning. Participants continued their normal life and responded to notifications which prompted them to complete state reports and the decision-making task over the next 2-wk. There were two notifications per day for 14 d between 09:00 to 13:00 and 14:00 to 18:00 UK time, which participants knew to expect but were not informed of the exact arrival times to minimize expectation effects. The actual times were pseudorandomized in 30-min intervals. States were reported at all notifications and the decision-making game was played on alternate days (25% of notifications, total games = 8). The number of games in the morning and evening was counterbalanced (4 each) and the order (morning or afternoon) was otherwise random. Participants were reimbursed a base rate and received additional payment for notification response based on the number of assessments they completed (up to £25.25) and the number of coins (reward) they collected in all games (maximum 25% of total payment was performance based).

### Assessments.

#### Subjective states.

The state assessment included 6 items and occurred at all timepoints (additional sleep quality item in morning only). State motivation was the sum score of four motivation items (Fig. 1*A*), derived from Apathy Motivation Index (32) and validated in a prior independent sample (*SI Appendix*). Covariate items were happiness (“Right now, I feel happy”), fatigue (“Right now, I feel tired”), and sleep quality (“Last night, I slept well”). Participants endorsed all items on a 7-point Likert scale (totally untrue, mostly untrue, slightly untrue, neither true or untrue, slightly true, mostly true, totally true).

#### Trait and demographic characteristics.

At baseline, participants reported demographic characteristics. To assess traits relevant to motivation, participants completed several validated questionnaires in the order; Apathy Motivation Index (32), Apathy Evaluation Scale (51), Five-Factor Model Rating Form (52), Barratt Impulsivity Scale (53), Depression-Anxiety-Stress-21 (54), Snaith-Hamilton Pleasure Scale (55), and Multidimensional Fatigue Inventory (56).

#### Effort-based choice task.

The effort-based choice task (Fig. 1*B*) was adapted for smartphone use from a desktop version (34). The game is a two-alternative, forced choice, effort-based task built in JavaScript using Phaser3 (3.23.0, https://phaser.io/) and hosted online with Google Firebase.

Participants were informed that they were traveling through a strange land of rivers and streams. At points along their journey, they would be/required to power up their magic umbrella to fly across the water. At each crossing point, they choose between different routes. Routes offer varying coins (1-7) but require varying effort (20 to 80%). Participants select their route (no time limit) and exert effort by pressing a button on screen as fast as possible until a power bar is filled (10 s time-limit). Effort levels are button presses as percentages of the participant’s maximum which was calibrated at baseline and fixed for the remainder of the study (*SI Appendix*). The differences in reward and effort (Fig. 3*A*) were decorrelated (*rho* = 0.06, *P* = 0.81) to aid choice model parameter estimation. Trial order was randomized with each game load and there was also randomization of minor aesthetics (e.g., colors and background assets) to give the impression of novelty. The task consisted of 24 trials divided into 2 blocks (median completion = 6.5 min).

### Statistical Analyses.

Analyses were conducted with R version 4.1.0 in RStudio version 1.4.1717. Hierarchical generative modeling was conducted separately on a high-performance computer with R version 4.3.2 and models fitted in Stan version 2.33.1.

#### Data validation and exclusion criteria.

We conducted data validity checks before the analysis. In order to retain the maximum number of participants, we applied exclusion criterion first at the level of timepoints and subsequently at the level of participants. In other words, we allowed participants to perform poorly on a single day (e.g., fail attention check on game 2) but well on other days (e.g., pass attention checks on all other games). Full details of the exclusion criteria are provided (*SI Appendix*). The valid timepoints were 94% of the collected data. Twenty-six people were excluded from the analysis because they did not provide valid data from ≥70% of the total timepoints.

The included participants (N = 129, age = 32.4 ± 0.6 y (mean ± 1SEM), 52.7% female) represented 83% of the recruited sample which is similar to the average drop-out rate in ecological momentary assessment studies (57). The majority of included participants provided valid data at the majority of assessments [total valid datapoints mode = 28 (97%), mean = 26.4 ± 0.2 or 91%; Fig. 1*C*]. Trait apathy in the included sample was significantly lower than the excluded participants, indicating that this trait predicted poorer completion of the assessments, which limited the range of trait apathy in our sample (*SI Appendix*).

For the linear mixed effects and generative modeling of behavior, an additional 9 participants were excluded because they did not have a trait apathy estimate (incomplete questionnaire data, n = 2) or they provided only 2 or 3 valid games (missing task data were > 50%, n = 7). This left 120 participants for complete task-based analysis (*SI Appendix*, Fig. S2). Catch decision trials were excluded from all task-based analyses.

#### Subjective states.

State motivation was well distributed with minimal ceiling (where state was maximum, mean = 7% ± 0.1%) and floor effects (where state was zero, mean = 1% ± 0.1%). To determine the relationship between state motivation and the other states, we quantified within-person (repeated-measures) correlations by estimating the common regression slope for paired states across individuals [rmcorr package *R* (58)]. This method appropriately accounts for the nonindependence of the data. We calculated between-person correlations as the Spearman rank correlation between 15-d mean states (Fig. 2).

For all other analyses (unless otherwise specified), the state was decorrelated from between-person characteristics by normalizing within-person (z-score). State motivation did not significantly differ between the morning (mean = −0.01 ± 0.02) and the afternoon assessments (mean = 0 ± 0.01; paired *t*_(128)_ = −0.39, *P* = 0.7, mean difference = −0.01, 95% CI [−0.09, 0.06], *d* = 0.03). To compare state motivation to days of the week, we conducted an ANOVA on the participant mean values for each day. To simplify post hoc *t* tests to a single comparison, we compared state motivation for the first part of the week (Monday–Wednesday) to the second part (Thursday–Sunday) with paired sample *t* test.

#### Trait apathy.

Trait apathy is a multidimensional construct which comprises several related dimensions (32, 59). To capture this, we took a dimensional approach and derived a single apathy score through confirmatory factor analysis (CFA) of apathy-motivation (Apathy Evaluation Scale & Apathy Motivation Index), fatigue (Multidimensional Fatigue Inventory), conscientiousness, and extraversion questionnaire subscales (Five-Factor Model Rating Form). We used standardized RMS of residuals (SRMR) < 0.08 and comparative fit index (CFI) > 0.9 as indicating good model fit (60). The 1-factor confirmatory factor analysis was a good fit (SRMR = 0.05, CFI = 0.93), suggesting that these characteristics were well captured by a single apathy dimension. CFA was conducted on all participants with complete questionnaire data (N = 154; i.e., regardless of sufficient follow-up data). Since trait apathy scores were not normally distributed as indicated by a significant Shapiro–Wilk test (W = 0.97, *P* = 0.003), Spearman’s rank correlation was used in simple correlations with this measure (Fig. 2). For the assessment of trait depression and anxiety, we replicated this procedure with an independent CFA on a different set of questionnaire subscale scores known to reflect this wider trait (*SI Appendix*).

We tested whether trait apthy predicted the 15-d mean and SD of (unscaled) state motivation using general linear models (with covariates of age and reported sex). Although trait apathy predicted lower state mean, it was not significantly related to the likelihood of floor effects, as assessed by binomial mixed effects model (β_trait-apathy_ = 0.95, *P* = 0.29). We assessed whether the autocorrelation of (person-centered) state motivation was related to trait apathy by fitting a linear mixed effects model in which the second daily state was predicted by the first daily state, trait apathy and their interaction and included a random slope for each participant. No random intercept was included (person-centering of state motivation already controlled for baseline differences in motivation level). We also reported the autocorrelation of state motivation using a summary statistics approach in which we fit an autocorrelation model for each person separately up to lag-4 (using acf function of stats package, R). This provided a point-wise estimate of the state autocorrelation for each person and indicates the strength of decay in autocorrelation over time (Fig. 2*F*).

#### Effort-based choices.

To confirm that the task elicited effort discounting, we conducted ANOVA on the mean probability of choosing the harder option (for each game; rank transformed data which did not violate ANOVA assumptions) as a function of the difference in effort, the difference in reward, their interaction, and the impact of time (study day). To confirm that the probability of success (filling the power bar and receiving reward) was stable across the study, we conducted a binomial regression with the dependent variable proportion of successful trials with a single predictor as time (day). We also confirmed that the proportion of trials in which participants exceeded their estimated maximum effort was low, as an indicator of appropriate effort calibration (μ = 0.1, allowing for 500 ms reaction time after stimulus presentation).

Test–retest reliability was calculated as intraclass correlation coefficients (ICC) of the behavioral summary statistics: mean proportion of harder options chosen and response vigor (press lag). For 8 games in the study across 15 d, we calculated the ICC for every 2-game combination and reported the mean, SE, and 95% CI (Fig. 3*D*).

#### Relationship between behavior, states, and traits.

We assessed the relationship between self-reported variables (states and traits) and decision-making using linear mixed-effect models (61). The best-fitting model accounted for individual variability in choices with individual random intercepts and slopes for the effect of state (Eq. **1**).

[1] $${\mathrm{choice}}_{i,t}∼state_{i,t}\times trait_{i}+\left(state|i\right),$$

*i = individual, t = timepoint*

1. Behavioral linear mixed effects model formula to predict choices [mean (chosen effort % – unchosen effort %)] from fixed effects of state motivation, trait apathy person-specific (random) state slope (state|i) and a random intercept (i).

Model validity was assessed through inspection of approximate linearity, normality of residuals, and random effects, collinearity of predictors (variance inflation factor < 5) and the lack of influential outliers using the performance package (62). The proportion of variance explained by all effects in this model (conditional *R*^2^ = 0.8) was substantial according to ref. 63. The majority of this was due to the random effects since the variance explained by the fixed effects alone was weak (marginal *R*^2^ = 0.04). The individual (random) intercepts and slopes were decorrelated (correlation of random effects = 0.01). The model was a better fit than a linear model with no random effects [X^2^(1) = 819.12, *P* < 0.001, BF > 1,000] and a linear mixed effect model which had random intercepts only [X^2^(2) = 13.33, *P* = 0.001, BF = 0.96; *SI Appendix*]. The specificity of the state effect was assessed by comparing each alternative state (happiness, fatigue, sleep quality) with state motivation in the same model using likelihood ratio test.

We also used a logistic mixed effects model with the dependent variable as the harder option on every trial during the study to confirm that the state, trait, and state–trait interaction effects were robust while controlling for the large impact of the task reward and effort options. Subsequently, we repeated the analysis with the interaction between state motivation and the difference in reward and effort on each trial (only) to examine how state was related to choices as a function of reward and effort (Fig. 5 and *SI Appendix*). Response vigor was captured as the speed of button pressing. Specifically, the task measured the time lag between button presses (ms, where greater lag = reduced effort). For each trial, we took the mean lag between button presses and inverted this positively such that higher values represent greater vigor (vigor = max(mean(press lag)) – mean(press lag)). To assess the predictors of response vigor, we conducted a similar linear mixed effects model predicting vigor on each trial from state motivation, trait apathy, and their interaction.

#### Hierarchical modeling of state and choices.

Building on our previous work (34), we modeled the influence of the timeseries of state motivation on choices using a hierarchical Bayesian model (*SI Appendix*). Since this was our goal, we did not compare different subjective value computations (e.g., parabolic, hyperbolic) and focused on the simplest possible linear effort discounting model with two free choice parameters which we previously identified as the most parsimonious model of healthy participants choices in this game (34).

The model was jointly fit to both choices and state endorsements. In the choice model, the subjective value of each choice is a function of the difference between reward and effort, scaled by reward and effort sensitivity for that person, at that time (Eq. **2**). Choice values map directly onto probabilities using a Bernoulli transformation (Eq. **3**).

[2] $$v_{i,t,trial}={\mathrm{rewSens}}_{i,t}\times {\mathrm{reward}}_{\mathrm{trial}}-{\mathrm{effSens}}_{i,t}\times {\mathrm{effort}}_{\mathrm{trial}}.$$

2. Linear discount of effort from reward to generate a choice model for each trial.

[3] $$choice_{i,t,trial}∼bernoulli\_logit\left(v_{i,t,trial}\right).$$

3. Choice value is transformed to a choice using a Bernoulli transformation.

The individual parameters (e.g., *rewSens* for each individual, i, at each timepoint, t) were a linear function of trait apathy $\left(\beta_{\mathrm{trait}}\times apathy_{i}\right)$, an individual parameter offset $\left({\tilde{rewSens}}_{i,t}\right)$, and the modulation of state motivation $\left(\beta_{\mathrm{state}}\times \theta_{i,t}\right)$ (Eq. **4**). In the timeseries model which compared the effects of past and future states, we compared these in the same model post hoc by replacing the current state $\left(\beta_{\mathrm{stateR}}\times \theta_{i,t}\right)$ with past and future states ($\left(\beta_{stateR-1}\times \theta_{i,t-1}\right)+\left(\beta_{stateR+1}\times \theta_{i,t+1}\right)$). We also replicated the superiority of the prior state motivation on reward sensitivity up to 2 timepoints in the past (*SI Appendix*).

[4] $${\mathrm{rewSens}}_{i,t}=\left(\beta_{\mathrm{traitR}}\times apathy_{i}\right)+{\tilde{rewSens}}_{i,t}+\left(\beta_{\mathrm{stateR}}\times \theta_{i,t}\right),$$

$${\mathrm{effSens}}_{i,t}=\left(\beta_{\mathrm{traitE}}\times apathy_{i}\right)+{\tilde{effSens}}_{i,t}+\left(\beta_{\mathrm{stateE}}\times \theta_{i,t}\right).$$

4. Latent subjective state motivation *θ* was allowed to independently influence reward sensitivity and effort sensitivity on a given day. The effect of state motivation (*θ*) on each parameter was quantified with the β weights which were given weakly informative priors centered on zero $\beta_{\mathrm{state}}∼normal\left(0,1\right).$ Trait apathy scores ($apathy_{i}$) independently influenced each parameter as an intercept with the specific effect scaled by independent β weights ($\beta_{\mathrm{traitR}},\beta_{traitE}$). *θ* was estimated independently in the hierarchical state model according to Eq. **5**. i = individual, t = timepoint.

To estimate the latent state *θ*, state motivation items (Fig. 1*A*) were binarized to agreement (any “true” response) or disagreement (all other responses). We fit a 1-parameter Item Response Theory model (64) to the state endorsements across all timepoints. The item difficulties (δ) were given a weakly informative prior [normal(0,1)] and minimum value of 0. This derived a (person × timepoint) matrix of latent state *θ* (Eq. **5**) indicating the likelihood of endorsing the motivation at that time. The individual *θ* was a linear combination of the group mean and participant level offset (Eq. **6**) and *θ* was normalized within-person (z-score) to decorrelate this parameter from trait apathy. State *θ* was highly correlated with the observed state (median Pearson’s *r* across participants = 0.85, *SI Appendix*, Fig. S8) indicating excellent posterior predictive accuracy. The (unnormalized) model-derived state *θ* was also strongly related to trait apathy (according to 15-d mean, *rho* = 0.63, *P* < 0.001). The probability of endorsing the state and making a choice was jointly fit to the participant’s choices and state endorsements using Bernoulli transformations.

[5] $$P_{n,t}={\mathrm{logit}}^{-1}\left(\theta_{j\left[n\right],t}-\delta_{i\left[n\right],t}\right),$$

$$Y_{n,t}=bernoulli\_logit∼\left(P_{n,t}\right).$$

5. Hierarchical item-response theory model with 1 subject free parameter. (P_{n, t})) is the likelihood that an item (n) at time (t) is endorsed, based on the subject's latent state θ and the item's difficulty δ. The responses ((Y_{n, t})) are then modeled as realizations from a Bernoulli distribution, where the probability parameter is defined by the computed likelihood of endorsement. j = subject index, i = item index.

[6] $$\theta_{i,t}=\theta_{\mu_{t}}+\tilde{\theta_{i,t}}.$$

6. Latent state motivation for individual, i and timepoint, t$\left(\theta_{i,t}\right)$ was estimated as an individual offset $\tilde{{(\theta }_{i,t}})$ from the group mean for that timepoint $\left(\theta_{\mu_{t}}\right)$. If individual data were missing for that timepoint, the parameter value was simply the group timepoint mean with no individual offset ($\theta_{i,t}=\theta_{\mu_{t}}$).

Following (34), we quantified the state effects [β_stateR_ and β_stateE_ (Eq. **4**)] at the group-level as 90% credible intervals using the highest density interval method (65). Posterior β estimates with 90% credible intervals that excluded zero indicated a meaningful modulation of the state on the choice parameter at a given time. For clarity, we also report the probability of direction (PD). PD is the proportion of the posterior with the same sign as the median, representing the likelihood of the existence of an effect (27, 39).

The posterior predictive accuracy of the model was excellent for choices (mean ± SEM = 0.91 ± 0.002) and subjective state endorsements (0.83 ± 0.01; *SI Appendix*). We also confirmed that the test–retest reliability of individual parameters was high (across all possible 2-game combinations, reward sensitivity = 0.93, 95% CI [0.91, 0.95], effort sensitivity = 0.76, 95% CI [0.73, 0.79]) and that the recoverability of individual parameters was excellent (Pearson’s *r* across all timepoints: reward sensitivity = 0.8, 95% CI [0.77, 0.82], effort sensitivity = 0.88, 95% CI [0.87, 0.9], state *θ* = 0.82, 95% CI [0.81, 0.83], *SI Appendix*).

## Supplementary Material

Appendix 01 (PDF)

## Data Availability

Anonymised data are available on Open Science Framework (https://osf.io/3mhqb/) (66). Code for all analyses and the smartphone game are available online (https://github.com/DevComPsy/reward-effort-2afc-firebase-EMA-motivation) (67).
