# Supplementary material for: Day-to-day fluctuations in motivation drive effort-based decision-making
Source: Proc Natl Acad Sci U S A. 2025 Mar 17;122(12):e2417964122. doi: 10.1073/pnas.2417964122 (PMC11962463; doi:10.1073/pnas.2417964122)
Supplement: Supplementary file 1 — Appendix 01 (PDF) [file pnas.2417964122.sapp.pdf]

Day-to-day fluctuations in motivation drive effort-based decision making

Supplementary Materials

Samuel RC Hewitt[1,2], Agnes Norbury[3], Quentin JM Huys[3], Tobias U Hauser[1,2,4,5]

[1] Max Planck UCL Centre for Computational Psychiatry and Ageing Research, Queen Square Institute of Neurology, University College London, UK

[2] Wellcome Centre for Human Neuroimaging, University College London, UK

[3]Applied Computational Psychiatry Lab, Mental Health Neuroscience Department, Division of Psychiatry and Max Planck UCL Centre for Computational Psychiatry and Ageing Research, Queen Square Institute of Neurology, University College London

[4]Department of Psychiatry and Psychotherapy, Faculty of Medicine, University Tuebingen, Tübingen, Germany

[5] German Center for Mental Health (DZPG), project site Tübingen

Contents

Data validation and exclusion criteria 3
State item validity 8
Task validity pilot 9
Day of the week effects 11
Maximum effort calibration 13
Trait apathy 14
Linear mixed effect modelling 15
State and trait effects as a function of reward and effort 17
Behavioural practice effects 19
Specificity of the state effect on choices 21
Specificity of the trait effect on choices 21
Hierarchical modelling of states and choices 23
Posterior predictive accuracy of the hierarchical generative model 24
Test re-test reliability of model parameters 25
Trait-apathy and reward/effort sensitivity 26
Temporal effects of state motivation on reward sensitivity 27
Supplementary Tables with Posterior Parameter Estimates 28
Supplementary References 31

Data validation and exclusion criteria

Several data quality checks were carried out prior to the analysis and specific data-points and participants were excluded if they failed to meet criteria below. If participants provided duplicate data at a given timepoint (e.g., by playing the game twice), we always took the first complete game and discarded the rest. The exclusion of timepoints were based on performance and attention checks (Supplementary Table 1). Firstly, we included two explicit attention checks in the subjective state report and decision-making task (game timepoints only). We also assumed that participants who completed the task very quickly or very slowly were not attending to it properly, and lastly we excluded data which was provided a long time after the notification was sent.

Timepoints which met these criteria (Supplementary Table 1) were marked as invalid (Supplementary Figure 1). Participants were then removed from the analysis listwise if they provided invalid data (according to above) at more than 30% of all study timepoints (>8 of 29). In total, 129 participants were included in the analysis (83%) who typically completed all the assessments (mode number of datapoints=28 (97%); Supplementary Figure 2). As reported in Results, 9 participants were additionally not included in the behavioural analysis only (linear mixed effects models linking decision-making and states and traits, and hierarchical Bayesian modelling) because they did not have complete trait-apathy data (n=2) or only completed 2 or 3 valid games (<50%, n=7). Therefore, the minimum number of games played was 4.

Of importance, this introduced a sampling bias where the excluded group had significantly higher trait-apathy at baseline (Wilcoxon rank sum W=1139, p=0.003) and the difference was considerable (cohen's d=0.65). This shows that people with the highest trait-apathy did exhibit this trait during the study, which was captured by reduced capacity to complete assessments (Supplementary Figure 3).

Order	Name	Criteria	N (%)
1	Attention	a) incorrect response to the attention check item. Participants were asked to agree with “Right now, I am completing an online study” for which we accepted “totally true” as correct *AND*<br>b) incorrect response on the catch decision-making trial. Catch trials offered an option with greater reward for less effort which we required participants to select, consistent with attentive responding and rational reward-effort decision-making following the approach in (1)	25 (2.3% of games)
2	Completion time	a) median choice reaction time for the game outside the 95% distribution across all games (1.19s - 6.67s) *OR*<br>b) time taken to complete the task outside the 95% distribution across all games (5.3 - 15.2 minutes)	103 (9.5% of games)
3	Response delay	Delay between scheduled notification time to response (across all games and self-report timepoints) > 240 minutes	73 (2% of all datapoints)

Supplementary Table 1. Attention and performance exclusion criteria applied to timepoints in the study. The criteria were applied in the order (Order) and excluded a total of 201 datapoints (6%). The effects of these criteria on reaction time and completion time distributions are plotted in Supplementary Figure 1. Participants that provided < 70% valid datapoints (according to these criteria) were excluded from all subsequent analyses (n = 31). An additional 2 participants were excluded because they did not provide complete questionnaire data for the assessment of trait-apathy and an additional 7 participants were excluded because their invalid data decision-making data exceeded valid data (i.e., they had < 50% valid games; Supplementary Figure 2).

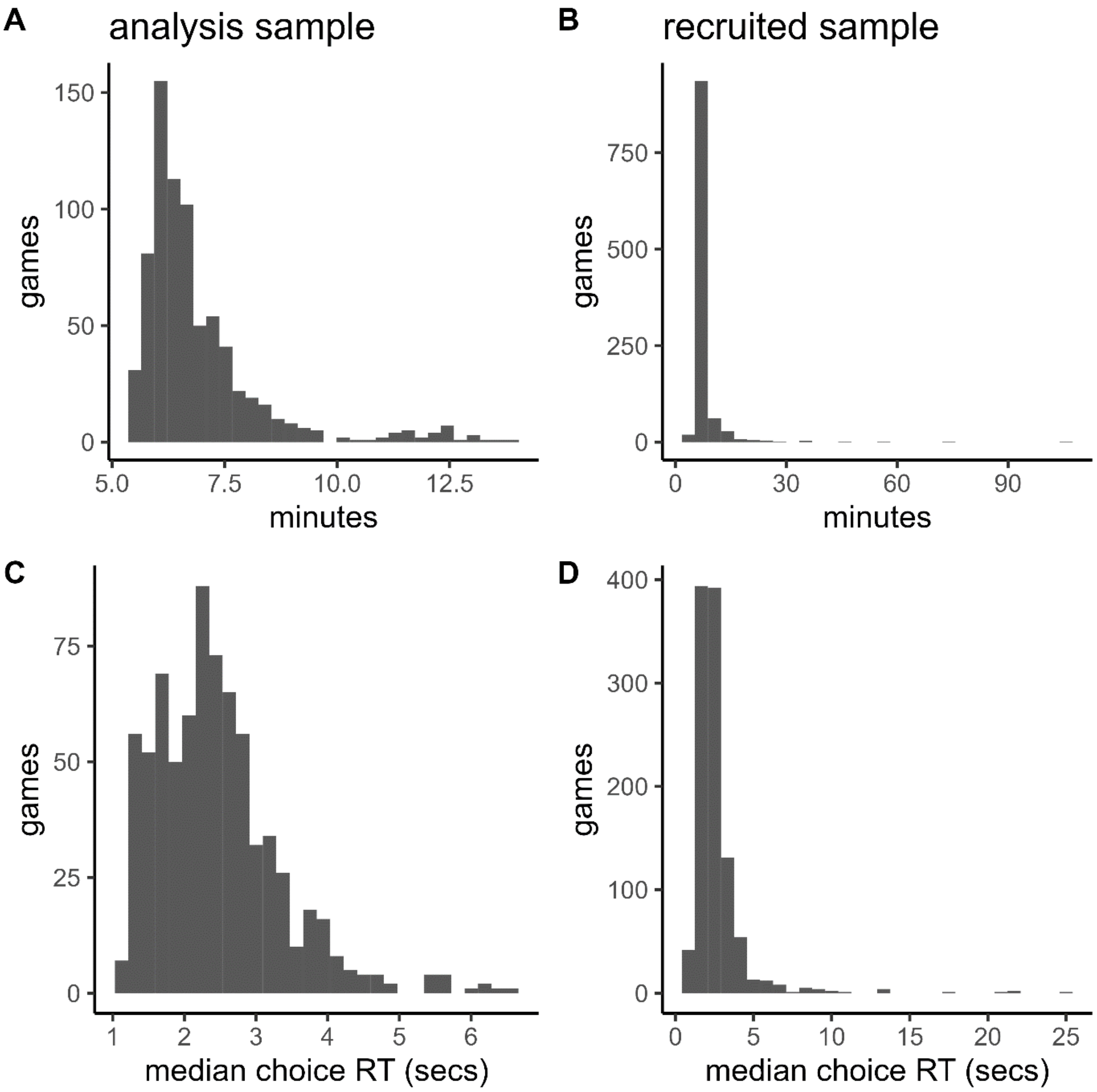

Supplementary Figure 1. Distributions of task completion times from start to end of each game (top, A&B) and median choice reaction times for each game (bottom, C&D) from the data that was included in the analysis (left, A & C) and the original, recruited sample prior to exclusion criteria (right, B&D). The exclusion criteria ensured that we included ~95% of games where participants passed validity checks and we did not include any games in which participants were very fast or very slow to complete the game as a whole and/or to make each decision.

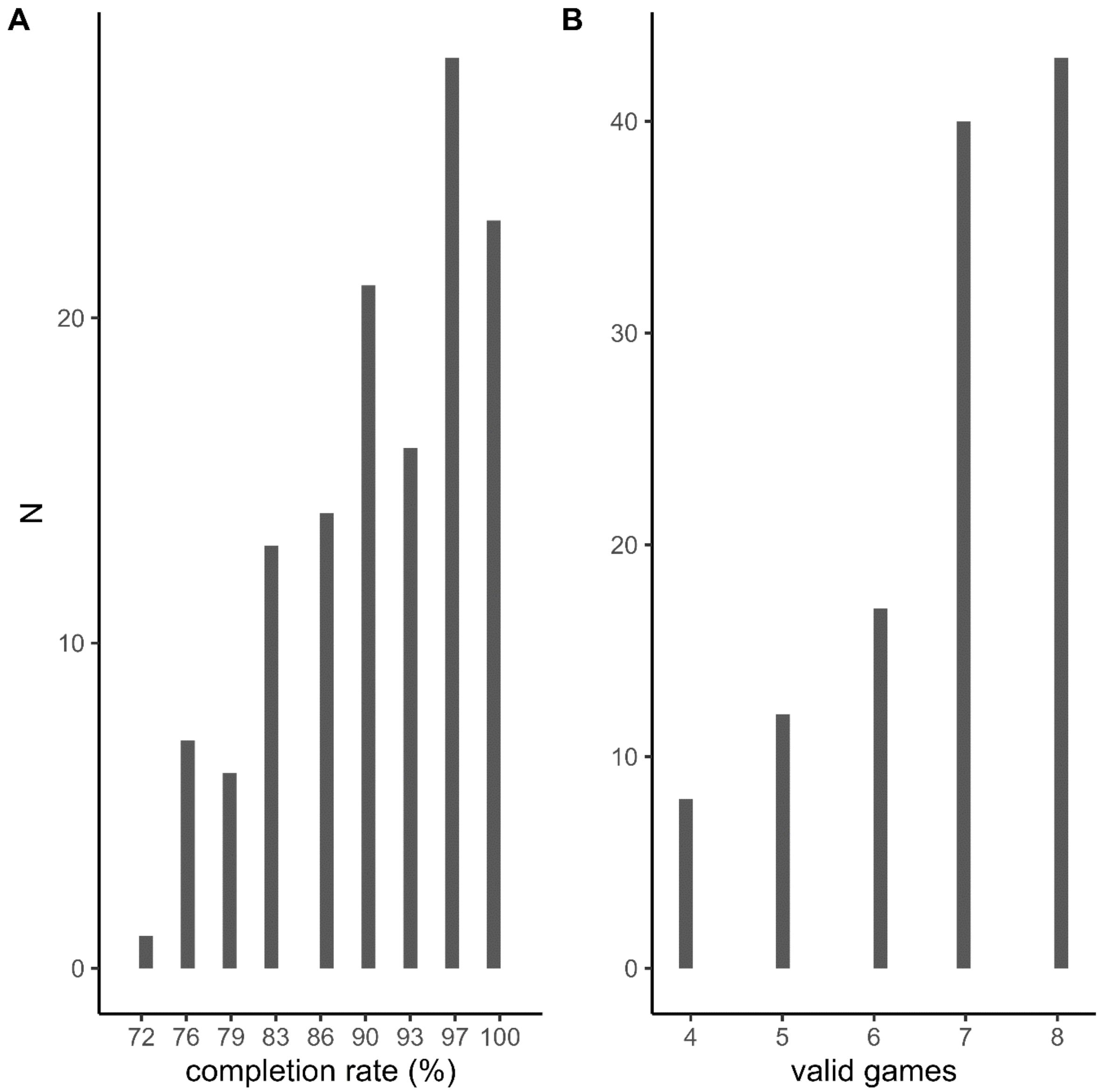

Supplementary Figure 2. Completion data for participants included in the analysis. A: The minimum number of datapoints (total across state and decision-making) from 129 participants was 21 and the maximum was 29 (2 per day for 14 days + 1 baseline). The mode number of datapoints was 28. For the decision-making specifically, we excluded 9 participants who only provided 2 or 3 valid games such that they had more invalid or missing data than they did which met validity checks. From the remaining 120 participants, the minimum number of games was 4 and the maximum was 8 (B).

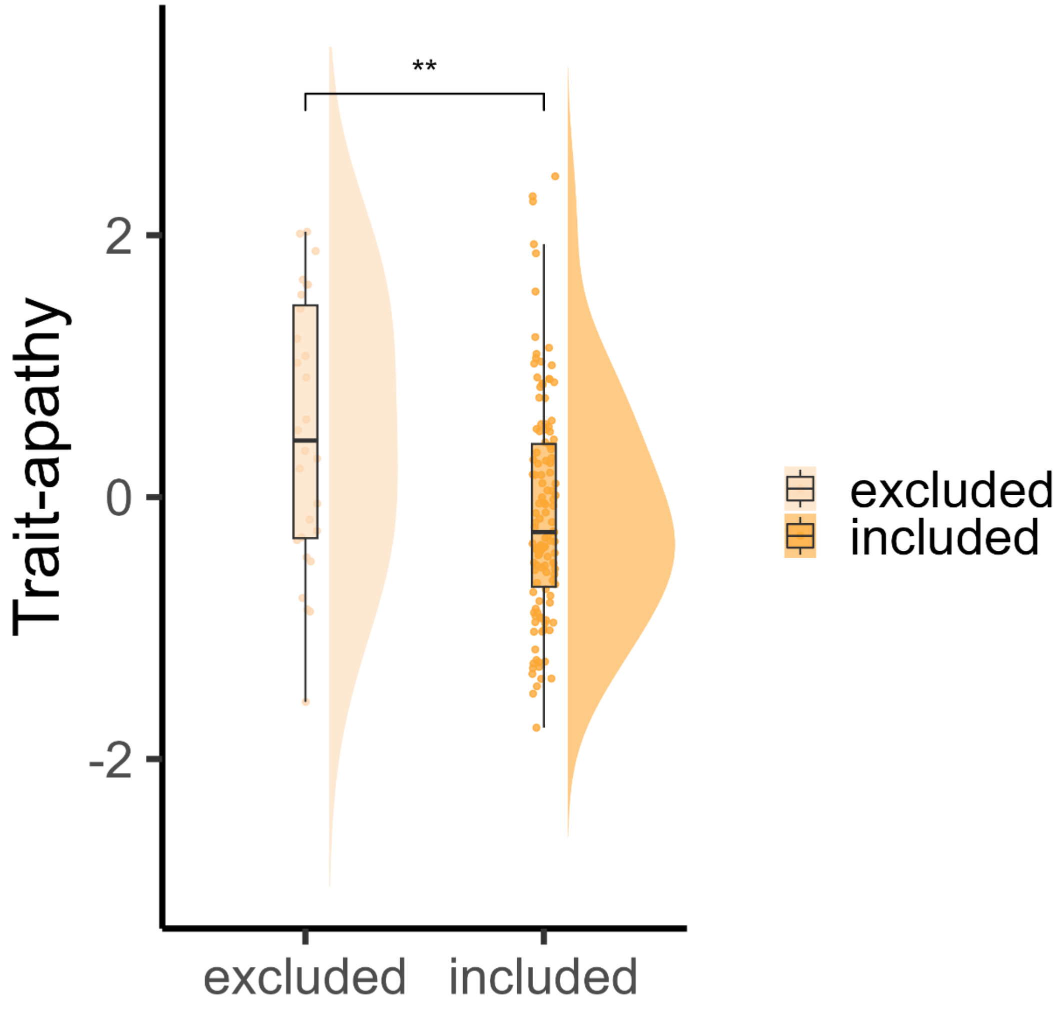

Supplementary Figure 3. Trait-apathy at baseline was significantly greater for the participants who failed to complete sufficient assessments, compared to those that were included in the analysis. This is in fact validation of the trait-apathy measure, given that it should predict reduced goal-directed behaviour (here quantified by study assessment completion). However, it does indicate that the design induced a sampling bias which is a limitation and should be carefully considered when designing future microlongitudinal studies. ** Wilcoxon signed rank non-parametric t-test $p < 0.01$

Attention checks

The self-reported attention check item was selected from the Comprehensive Infrequency/Frequency Item repository (2) based on recent recommendations to employ a frequency-item attention check over instructed-item checks (3–6). We reasoned that if the attention check item were to change at each timepoint (while all remaining questions did not change), then this would be suspicious and flag to participants that the item was a covert attention check. Therefore, we selected this single frequency item only (item 483). The item was selected from those in the database with high mean scores, based on its appropriateness for momentary assessment and objectively correct answer (Totally true).

The decision attention check (catch trial) was a single trial where a higher reward was offered for a lower effort level. Selecting the lower effort option was consistent with properly evaluating the options in a cost-benefit manner. Since the trial order was randomised on loading of each game, the catch trial was embedded at different points in the game on each play necessitating greater attention to detect it.

State item validity

Before the main study, we conducted a pilot to determine the feasibility of the study design and validate the state motivation items (n=40). This pilot contained self-report ecological momentary assessment items assessed twice per day for 2 weeks as in the full study reported in Results. Here, we also found that the majority of participants completed the majority (>70%, n=35) of the assessments. In this pilot, we found that state motivation was positively correlated with state happiness (between people: $rho(33)=0.49$, $p=0.003$; within-people: $r_{(1078)}=0.42$, 95% CI [0.37, 0.47], $p<0.001$) and sleep quality (between people: $rho=0.41$, $p=0.014$; within-people: $r_{(502)}=0.3$, 95% CI [0.22, 0.38], $p < 0.001$) which was very similar to what was found subsequently in the main study. This pilot included a state energy measure (“I feel energetic”), which was very strongly related to motivation between people ($rho(33)=0.81$, $p<0.001$; within-people: $r_{(1078)}=0.56$, 95% CI [0.52, 0.6], $p<0.001$). The strong correlation between self-reported energy and motivation suggested that energy was not distinguishable and not useful to retain, so we changed the direction of this item to fatigue “I feel tired” for the main study (reported in Results). We confirmed the autocorrelation finding that state motivation earlier in the day predicted state motivation later that day ($\beta_{state-1}=0.31$, $p<0.001$), which was also replicated in the main study (Results). Finally, state motivation was also negatively predicted by trait-apathy (Apathy Motivation Index: $rho(33)=-0.37$, $p=0.028$) and trait depression-anxiety (DASS21 total: $rho(33)=-0.49$, $p=0.003$) at baseline. For the main

study, we added additional trait questionnaires and took a transyndromal approach to summarise apathy across several related questionnaires (see Trait Apathy).

Task validity pilot

We also conducted pilot studies to confirm the validity and test re-test reliability of the task, prior to the main study. In the test re-test reliability pilot, 59 participants completed the game twice, 24-hours apart. The test re-test reliability of behaviour was moderate-high and very similar to what was later found again in the main study (see Results; intraclass correlation coefficients: P(harder option)=0.61; vigour=0.85). We also found that model-based reward and effort sensitivity could be reliably estimated over 24-hours (reward sensitivity = 0.64; effort sensitivity = 0.84) with this sample size. In this sample, motivation at the time of decision-making was positively related to willingness to make effort in the task (β=2.7, 95% CI [0.42, 4.99], p=0.019; (Supplementary Figure 4).

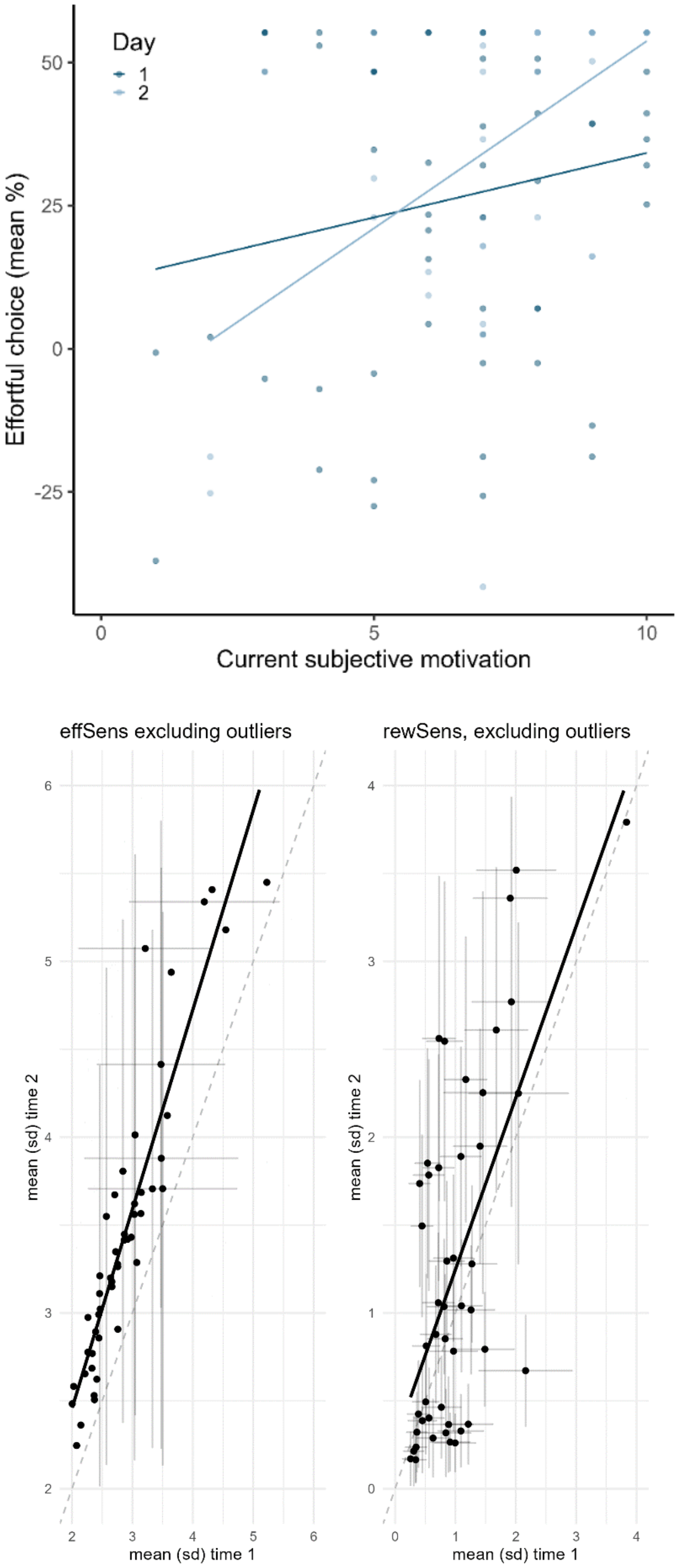

Supplementary Figure 4. Task pilot data. Top: relationship between current state motivation (x-axis) and willingness to make effort (y-axis, mean effort chosen - unchosen). Bottom: test re-test reliability of reward and effort sensitivity parameters estimated hierarchically over participants and timepoints (similar to the main results).

Day of the week effects

We found that (person-centred) state motivation fluctuated significantly across the week (main text, Figure 2). We also confirmed that this effect was not driven only by responses to the social motivation items by repeating the analysis for behavioural and social domains separately. This revealed that state motivation across both domains drove differences across the days of the week (behavioural: $F_{(6,985)} = 2.45$, $p = 0.022$, η2 = 0.01; social: $F_{(6,985)} = 3.91$, $p = 0.001$, η2 = 0.01). Although social motivation peaked more on the weekend (Friday-Sunday; Supplementary Figure 5) and marginally did not actually significantly differ between Mon-Wed and Thurs-Sun (paired $t_{(128)} = -1.88$, $p = 0.06$, mean difference = -0.08, 95% CI [-0.17, 0]; d = 0.17). However the effect sizes for the difference between Mon-Wed and Thurs-Sun were in a similar range for both behavioural and social motivation items (behavioural $d = 0.25$, social $d = 0.17$).

This reassured us that the fluctuations across the week were not due only to changes in social motivation (but were similarly driven by the two behavioural items as well). It is important to note that to simplify the post-hoc comparisons between days, we arbitrarily split the week as Mon-Weds and Thurs-Sunday, and the pattern may well differ if this split was adjusted. Importantly, the effect sizes for all between-person differences are small, leaving substantial room for individual variability. This is evident for both measures, where some people exhibit the opposite weekly fluctuations, with greater motivation earlier in the week which decreases in the second part (Supplementary Figure 5).

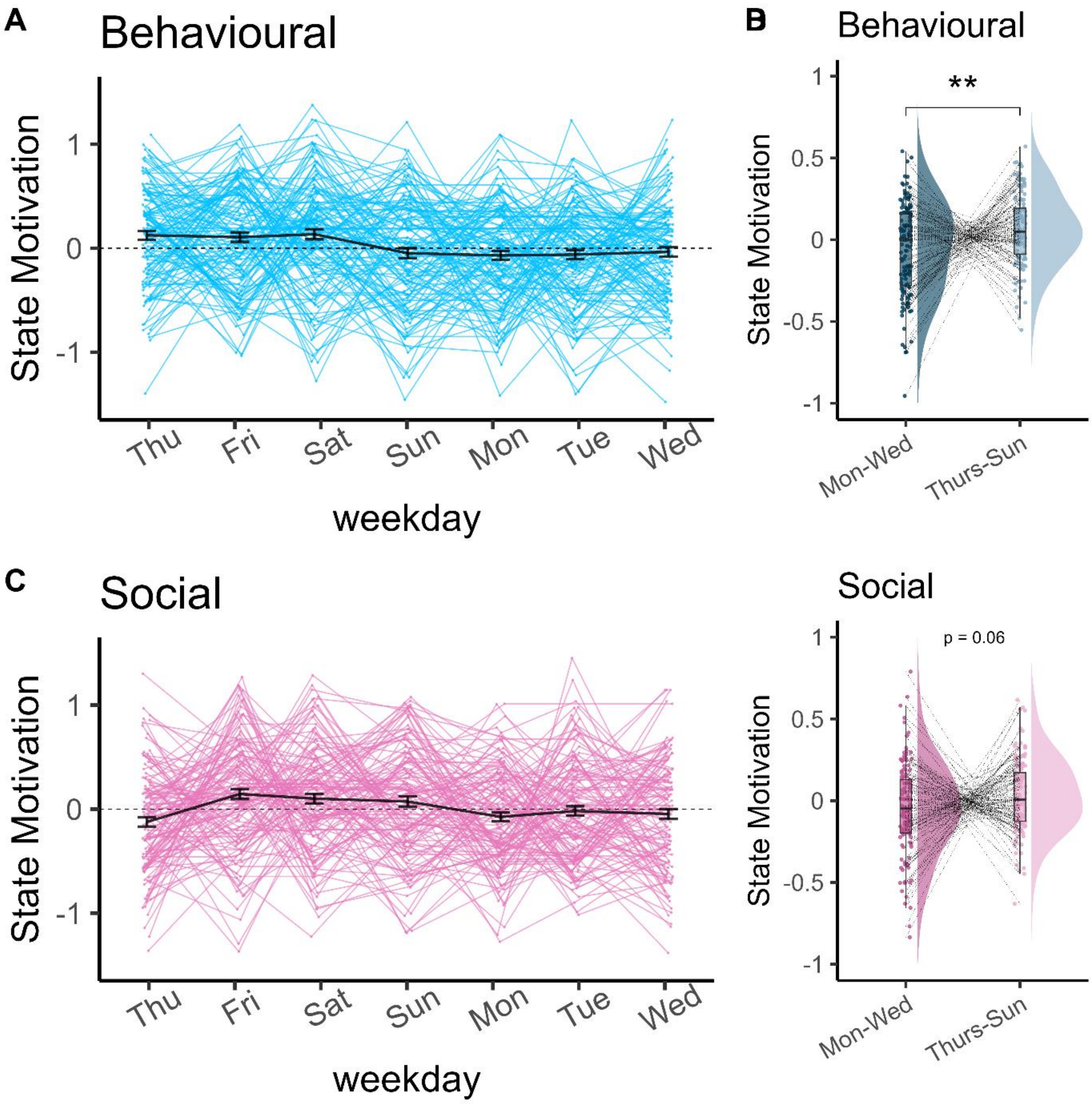

Supplementary Figure 5. Fluctuations in state motivation across days of the week for behavioural (A,B) and social motivation (C,D) separately. **paired t-test *p* = 0.005

Maximum effort calibration

Effort for each task option in the mobile game is represented as a proportion of the participant's maximum number of button presses within 10-seconds (100% effort). The maximum effort estimate was calibrated at baseline similar to previously validated tasks (1, 11–13) and fixed for the remainder of the two-week study.

The specific calibration procedure was as follows; participant's 100% effort level was initialised at baseline as the maximum presses across a practice phase of 3 trials. In the practice phase, the amount of presses required to fill the bar was set at a level which < 1% of pilot participants were able to achieve so that participants were nudged to exert as much as they could (they were unlikely to successfully fill the bar). We did not explicitly tell participants to exert their maximum effort to avoid disclosing this demand characteristic to attentive participants. This is advantageous because seasoned participants might deliberately "game" this instruction knowing that exerting less than their true maximum might be beneficial to them. As an additional guard against this, we set a minimum initial 100% effort level of 58 button presses which was based on prior piloting. Next and undisclosed to participants, a recalibration phase occurred in the first two trials of the baseline game (day 0 only). In this recalibration phase, if the effort exertion time was less than the expected time given the chosen effort (e.g., 80% effort should take ≥ 80% of the time i.e., 8 seconds), the participant's maximum effort level was recalibrated in line with this (supplementary equation 1). The 100% effort level was only ever *increased* by recalibration (never decreased from the initialised value). The mode number of recalibrations was 1 (max 2). After the recalibration phase, the 100% effort level was fixed for the remainder of the 14-day study and retrieved from the cloud database when the participant loaded the task.

$$\text{maximum effort (presses)} = \left(\frac{press\ count}{press\ time\ (s)}\right) *\ 10(s)$$

Supplementary Equation 1. Maximum effort threshold formula applied on recalibration trials 1 and 2 (baseline game only) if participants successfully filled the power bar faster than the proportion of time predicted by their chosen effort level. This formula was also used to calculate the estimated maximum effort (Results, Figure 3B).

The estimated maximum effort calibrated using this procedure was stable across the study (see Results). We also calculated the test retest reliability (intraclass correlation coefficient) of participant's maximum effort within 10-seconds based on the formula (Supplementary Equation 1) and found this to be very good for the duration of the study (ICC day0 to day14 = 0.79).

Trait apathy

For the multidimensional trait apathy score, we included Apathy Evaluation Scale total, Apathy-Motivation Index total, Five-Factor Model Rating Form extraversion, Five-Factor Model Rating Form conscientiousness and Multidimesional Fatigue Inventory total scores because of their conceptual and empirical link. Both trait extraversion and trait conscientiousness were also associated with trait apathy scores from the Apathy Evaluation Scale and Apathy Motivation Index (range rho [-0.57, -0.49], Supplementary figure 6). We also confirmed that 1-factor was suitable for these dimensions using the modified procedure of Horn's Parallel analysis (14–16), which determines whether an eigenvalue is larger than what could be expected by chance.

Consistent with our expectations, we found that state motivation as measured day-to-day was independently strongly related to trait extraversion (mean state motivation: β=0.48, *p*<0.001; mean behavioural items only: β=0.17, *p*<0.001, mean social items only: β=0.31, *p*<0.001) and trait conscientiousness (15-day mean state motivation: β=0.41, *p*<0.001; behavioural items only: β=0.23, *p*<0.001, social items only: β=-0.18, *p*=0.003).

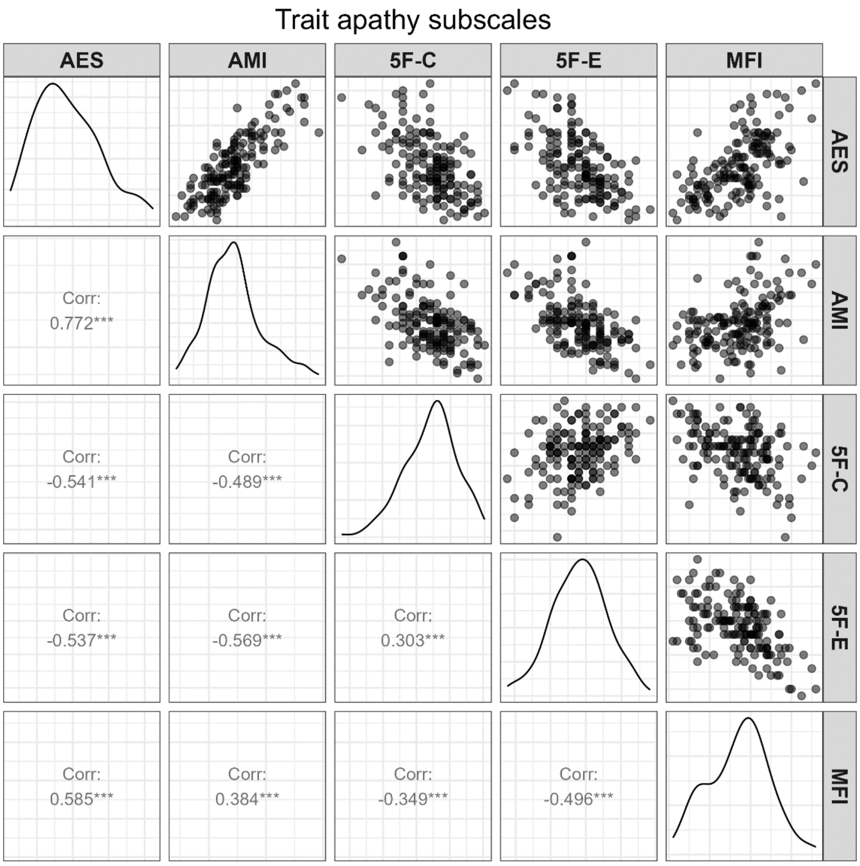

Supplementary Figure 6. Pairplot of the subscales in trait apathy dimensional score (AES=Apathy Evaluation Scale Total, AMI=Apathy-Motivation Index Total, 5F-C=Five-Factor Model Rating Form-Conscientiousness, 5F-E = Five-Factor Model Rating Form-Extraversion, MFI=Multidimensional Fatigue Inventory.

Linear mixed effect modelling

We compared a limited number of linear mixed effects models which are summarised below (Supplementary Table 2). Conditional effects and the residuals of the best fitting model (model 4) are shown below (Supplementary Figure 7).

Behavioural model comparison

model	AIC	BIC	RMSE	formula
1	7,135.98	7,168.92	18.81	choice ~ state*trait + Age + Sex
2	6,332.12	6,369.78	8.45	choice ~ state*trait + Age + Sex + (1|i)
3	6,328.19	6,356.43	8.45	choice ~ state*trait + (1|i)
4	6,318.86	6,356.51	7.87	**choice ~ state*trait + (1+state|i)**

Supplementary Table 2. Model comparison indices for behavioural models predicting willingness to make effort (best=bold). The dependent variable, mean choice (%) is the mean (chosen effort - unchosen effort) for that person, on that day.

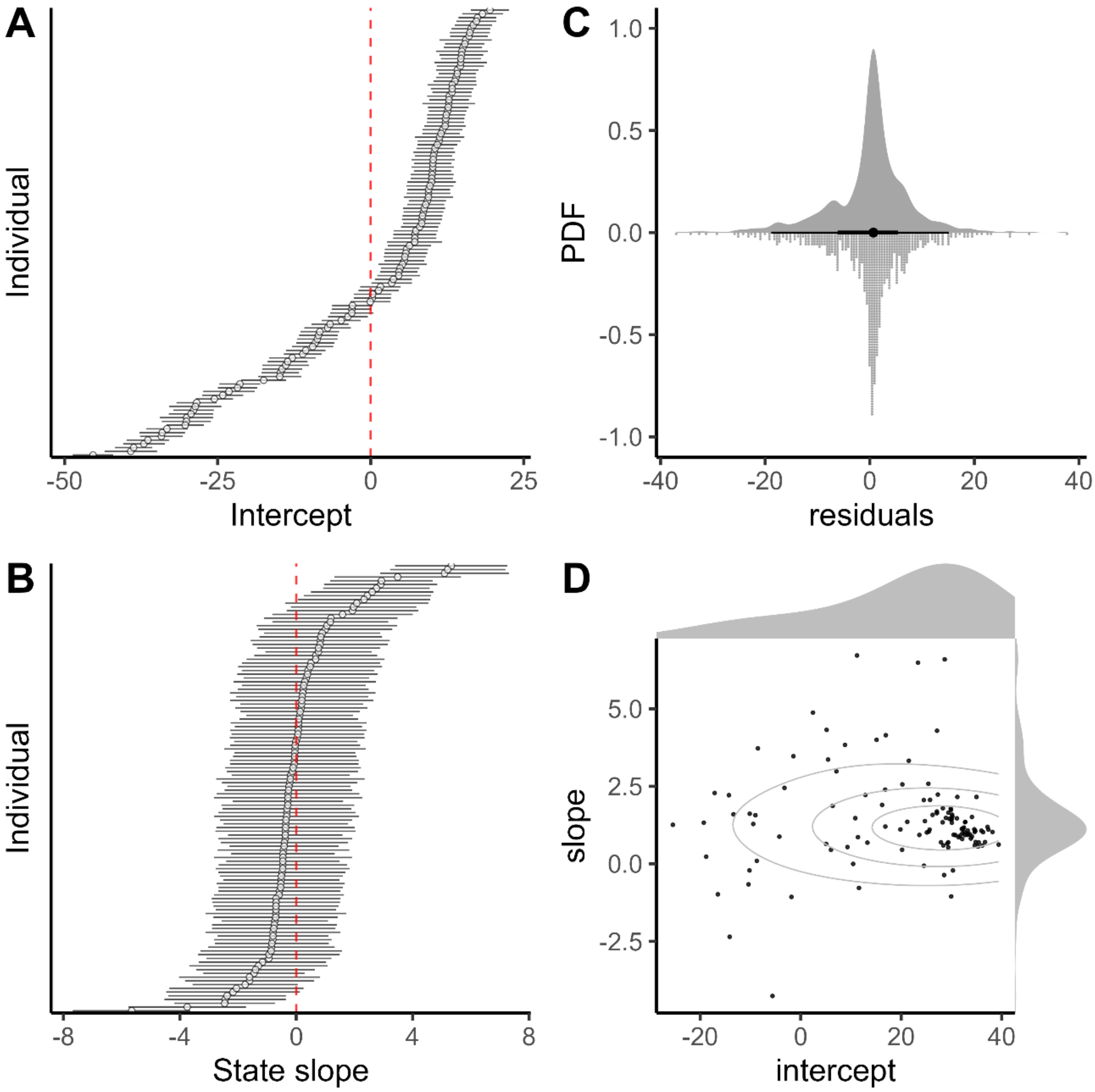

Supplementary Figure 7. Conditional random effects and residuals from the behavioural linear mixed effects model reported in Results. Conditional refers to the fact that these effects are conditional on the all other effects in the model. A: Conditional random intercepts estimates ± their standard deviation for each participant in the study with reference line on zero (red). B: Conditional random slopes ± their standard deviation. C: Approximate normality of residuals of the model. D: No correlation between the conditional slopes (y-axis) and conditional intercepts (x-axis).

State and trait effects as a function of reward and effort

To visualise the modulation of state on P(harder option) at each level of reward and effort, we ran a linear regression model (similar to Chong et al., 2017) to calculate the estimate marginal P(harder option) mean at each level of reward and effort when the state was low (person mean -2SD) and high (person mean +2SD) on each day (emmeans package, R). Following Chong et al., 2017, we conducted a separate model to test the interaction between state motivation and difference in effort on P(harder option) and we also separately tested the effects of trait apathy (on subject level means across the entire set of tasks)

As a function of differences in reward, state motivation significantly increased P(harder option) when the absolute difference in reward was low (deltaReward=1, β=0.13±0.03, p<0.001) and medium (delta reward=2, β=0.09±0.03, *p*<0.001) but not when the difference was largest (delta reward=3: β=0.05±0.03, *p* = 0.07, Supplementary Figure 8A). As a function of differences in effort, the trend was similar but state motivation significantly increased P(harder option) at all levels (+20%: β=0.06±0.03, p=0.028; +40%: β=0.07±0.03, p=0.006; +60%: β=0.14±0.03, *p*<0.001, Supplementary Figure 8B).

We also confirmed whether trait apathy had the same effect on choices as a function of reward and effort by replacing the modulation of state motivation with trait apathy score. As might be expected from a time-invariant (between person) predictor which necessarily has a fixed effect across all games, this revealed a uninform (value-independent) effect on P(harder option) at all levels of reward and effort (all p < 0.01). In other words trait-apathy on average, was associated with significantly reduced P(harder option) regardless of reward and effort difference (Supplementary Figure 8C-D).

This model agnostic analysis shows that trait apathy was associated with a strong reduction in P(harder option) reflecting a general tendency to decline the harder option regardless of value. In contrast, state motivation was associated with robustly increased willingness to make effort at low-medium reward differences only (not when the difference was high). For effort, state was associated with increased willingness at all levels. This

suggests that state motivation was more strongly associated with sensitivity to reward value. We next formally investigated this in the hierarchical generative model.

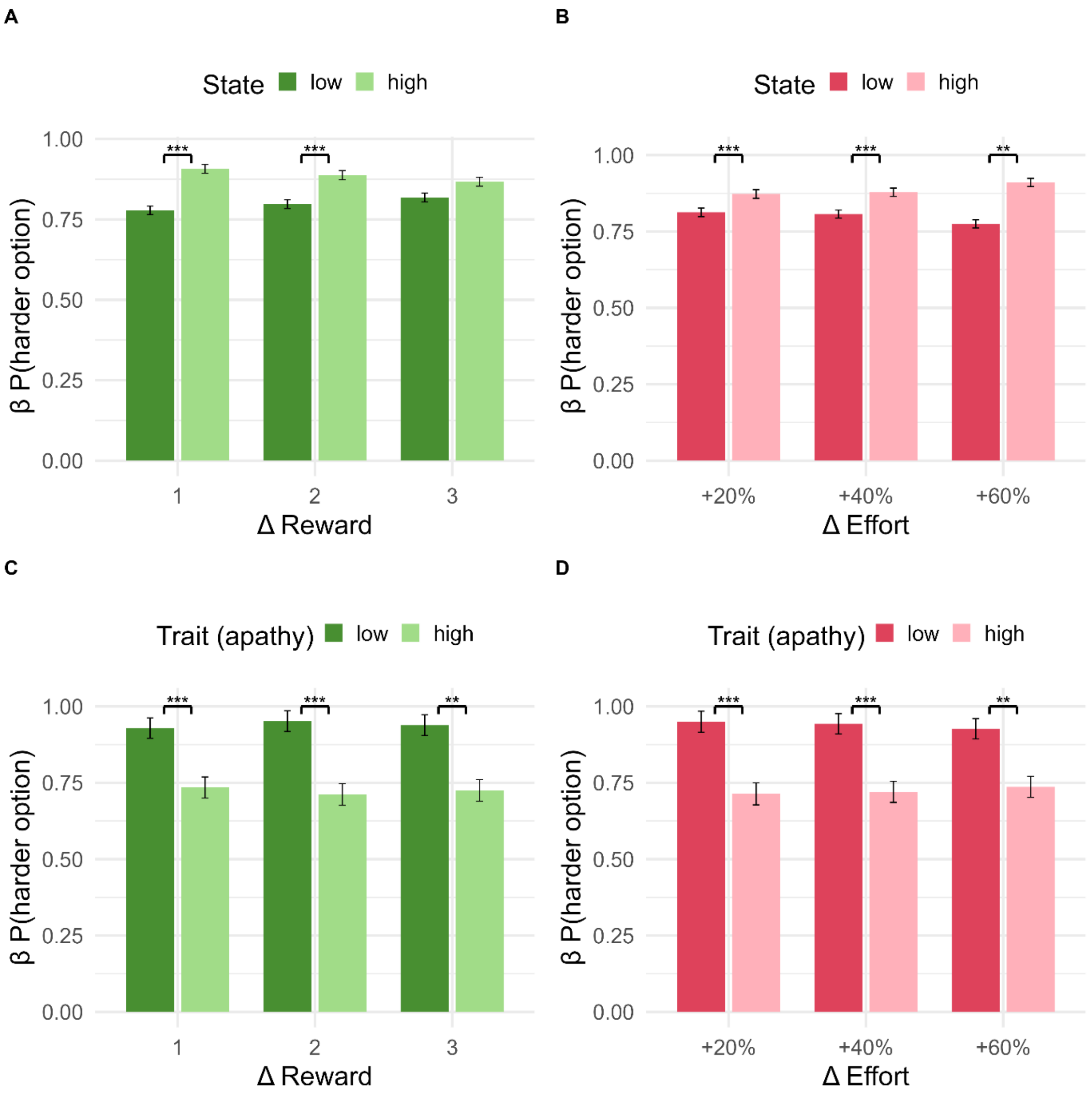

Supplementary Figure 8. Effect of state (top) and trait apathy (bottom) on the estimated marginal mean P(harder option) as a function of absolute difference in reward (left) and difference in effort (right, see also Figure 3A main text). *p<0.05, *p< 0.01, *p<0.001 for Low state/trait=-2SD (left bar, darker colour), high state/trait= +2SD (right bar, lighter colour). Trait apathy is normalised between people and the estimated marginal means are at the level of each subject (between person). State motivation is normalised within people and the estimated marginal means are at the level of each game.

Behavioural practice effects

To determine the effect of practice on behaviour, we fit a power-law curve linear regression to each person's choices separately. Power-law curves are a classic model of practice effects (17), which in our case would represent increasing willingness to make effort for reward over the first few sessions, until this stabilises and remains similar for remaining sessions. In this study, the mean proportion of variance explained across all participants by a power-law model was moderate (mean R^2 = 0.34, 95% CI [0.29, 0.39]) indicating that practice effects did contribute to the choice data. However, the proportion of variance explained was low compared to a recent study which tested the impact of practice across several tasks (17). The addition of subjective state to the model across subjects (mean R^2 = 0.47, 95% CI [0.42, 0.51]) significantly increased the proportion of variance explained compared with practice effects alone (paired $t_{(115)} = 8.14$, $p < 0.001$, mean difference in R^2 = 0.13, 95% CI [0.1, 0.16]; Supplementary Table 3). This indicates that although both state and practice effects were present (Supplementary Figure 9), practice alone was not a sufficient explanation for participant's choices.

model	mean	SEM	2.5% CI	97.5% CI	formula
practice	0.34	0.02	0.29	0.39	log(choice) ~ log(day)
practice + state	0.47	0.02	0.42	0.51	log(choice) ~ log(day) + log(State)

Supplementary Table 3. Comparison of linear regression model predicting choices from practice over days compared with the additional effects of subjective state motivation (state). Although practice effects were moderate, subjective states explained a significantly greater proportion of the variance in choice behaviour across people in this study.

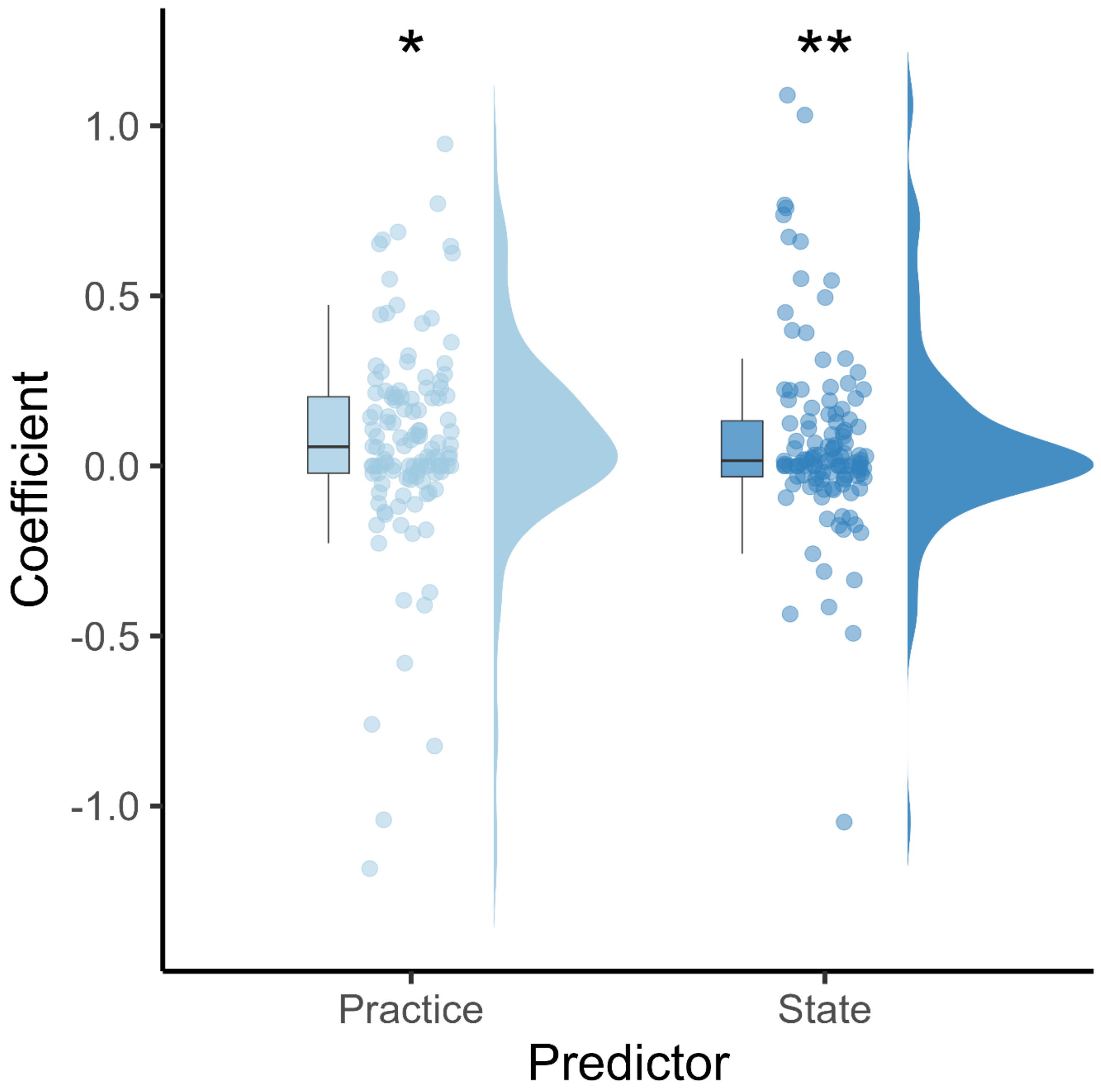

Supplementary Figure 9. Coefficients for practice and state effects on choices as estimated using a simple linear regression for each person independently. *independent t-test $p < 0.05$, **independent t-test $p < 0.01$

Specificity of the state effect on choices

The specificity of the state motivation effect on choices was assessed by comparing each alternative state (happiness, fatigue, sleep quality) with state motivation in the same model using likelihood ratio test. Sleep quality was only reported in morning, so the report was filled to afternoon timepoints on that day if the task was completed at the second notification.

State motivation was a superior predictor of choice than state happiness (see Results) and also state fatigue ($\beta_{fatigue}$ = -0.29, 95% CI [-0.98, 0.41], p = 0.42; improved fit with state motivation: $X^2_{(1)}$ = 8.69, p = 0.003; Figure 4C) and sleep quality (β_{sleep} = -0.15, 95% CI [-0.85, 0.55], p = 0.67; improved fit with state motivation: $X^2_{(1)}$ = 11.79, p < 0.001).

Specificity of the trait effect on choices

We also confirmed that the state-trait interaction was specific to apathy by comparing the effect of trait depression and anxiety. To summarise trait depression and anxiety, we replicated the CFA procedure that was used to estimate trait-apathy with an independent CFA on a different set of questionnaire subscale scores known to reflect this wider trait. These were Depression, Anxiety and Stress subscales of the Depression-Anxiety-Stress scale (18), neuroticism (of Five-Factor Model Rating Form, Mullins-Sweatt et al., 2006) and anhedonia as measured by Snaith-Hamilton Pleasure Scale (20). This 1-factor confirmatory factor analysis was a good fit to these questionnaire scores (SRMR = 0.03, CFI = 0.99), showing that these characteristics were also well captured by a single dimension. We confirmed the construct validity by comparing to 15-day mean state happiness and trait-apathy scores (Supplementary Material). In contrast, the subscales which comprised both the trait-apathy and depression-anxiety dimensions were not well fit to a single factor according to SRMR > 0.08 and CFI < 0.9 (observed SRMR = 0.14, CFI = 0.61).

Despite being positively related to trait-apathy (rho = 0.4, p < 0.001), trait anxiety-depression did not predict choices in general (main effect $\beta_{trait\text{-}depression}$ = -0.36, 95% CI [-4.41, 3.69], p = 0.86) and was not significantly related to the coupling between states and choices (i.e., no significant interaction with state motivation; $\beta_{State*trait\text{-}depression}$ = 0.55, 95% CI [-0.58, 1.69], p = 0.34). This shows that the coupling between state motivation and choices was specifically modulated by motivational (not affective) traits, in line with previous work in healthy people (21).

We estimated depression and anxiety traits, similar to the approach for trait-apathy as reported in Results (Figure 2 & 4). Like trait-apathy scores, trait depression-anxiety scores were not normally distributed according to significant Shapriro-Wilk test (W = 0.94, $p < 0.001$) so we compared these with alternative measures using Spearman's rank correlation. We would expect depression-anxiety scores to negatively predict mean happiness (as assessed by endorsement of the ecological momentary assessment item "Right now, I feel happy") across the 15 days in the study which was indeed the case (*rho* = -0.41, *p* = < 0.001). Depression-anxiety scores were also positively correlated with trait apathy scores (*rho* = 0.4, $p < 0.001$, Supplementary Figure 10) as is usually the case in studies which assess both (7, 22–25).

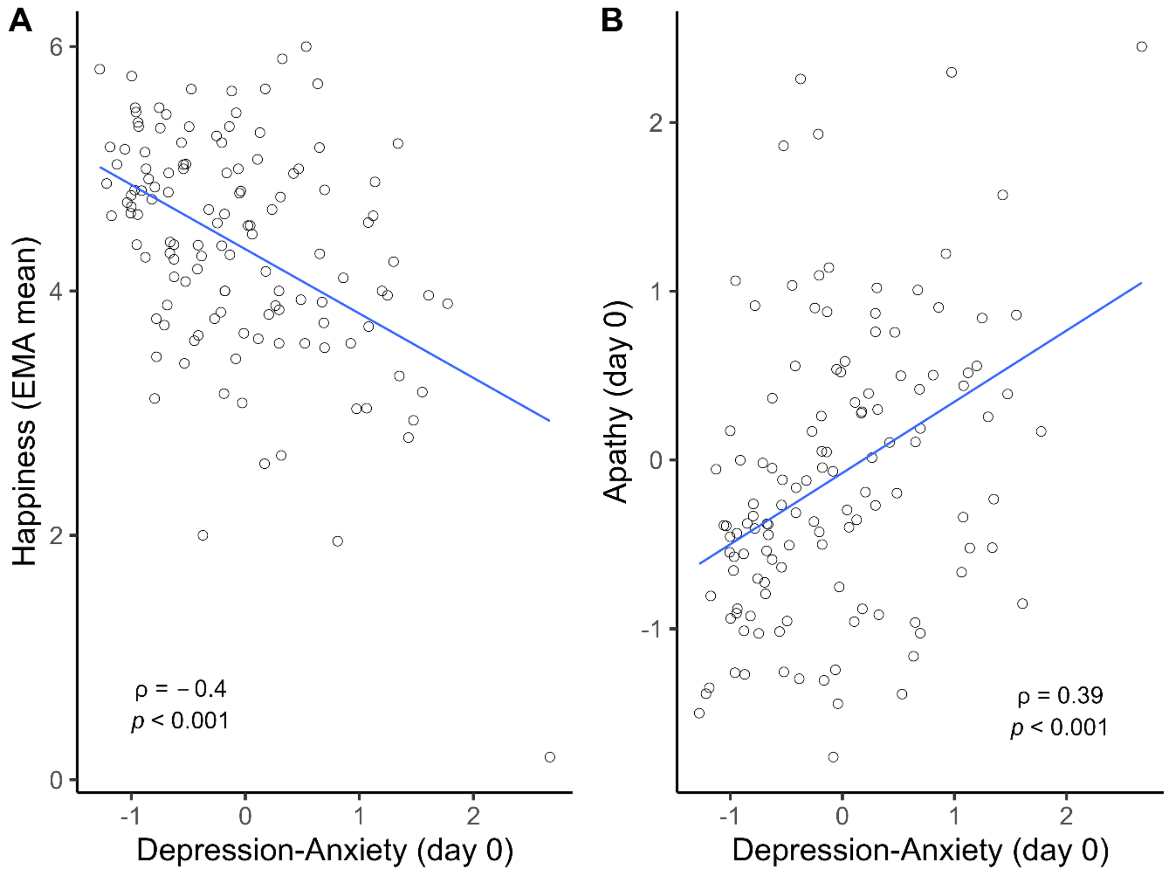

Supplementary Figure 10. Construct validity of the depression-anxiety score derived through confirmatory factor analysis of the Depression-Anxiety-Stress Scale, Neuroticism (Five-Factor Model) and anhedonia (Snaith-Hamilton Pleasure Scale). A: Depression was moderately negatively correlated with mean happiness in the subsequent 2 weeks as we would expect (EMA=ecological momentary assessment). B: Depression was moderately positively correlated with trait-apathy at baseline.

We also examined whether trait impulsivity was related to effort-based choices and the coupling between state motivation and choices. Trait impulsivity might be an important covariate because it is positively related to both apathy (26) and behavioural variability (27). Impulsivity (as indexed by Barratt-Impulsiveness Scale Total Score) was not significantly related to effort based choices (main effect β=-1.36, 95% CI [-4.37, 1.64], *p*=0.38) and was not significantly related to the coupling between states and choices (i.e., no significant interaction with state motivation; β=0.01, 95% CI [-0.86, 0.88], *p*=0.98).

Hierarchical modelling of states and choices

Choices and states were jointly modelled using a hierarchical Bayesian model. This approach improves parameter estimation compared with methods which fit independent models for participants or timepoints (17, 28), This is also advantageous because we explicitly modelled our expectations about the link between the population-level and the individual, and embed statistical inference directly into parameter estimation (rather than post-hoc).

Model fitting followed best practice recommendations for Bayesian generative models (29, 30). We used cmdstanr version 2.33.1 (31) to implement Markov-Chain Monte Carlo (MCMC) sampling of model parameters in Stan. MCMC chains were fitted with random starting values range [-1.25, 1.25] with 4000 samples (2000 warm-up). The convergence of 4 parallel chains was confirmed with $\hat{R}$ < 1.01 for all parameters, indicating excellent convergence (32). For each parameter, the ratio of effective samples to the total available was sufficient (mean = 3.2, range [0.5, 6.2]) and all chains had zero divergent transitions. All group parameters were given generic, weakly informative priors (equation S2) following (1).

$$\mu \sim N(0,1)$$

$$\sigma \sim Cauchy(0,1)$$

$$R_{chol} \sim LKJ(1)$$

$$\beta \sim N(0,1)$$

S2. All group level parameters were given weakly informative priors. σ had a minimum bound of zero. All other parameters were unbounded.

Group level parameters (mean and SDs) were derived from a covariance matrix to maximally pool information across all the available timepoints (equation S3).

$$rewSens_t \sim MVNormal\left(\left[\text{rewSens}_{\mu,t}\right], \sigma_{rewSens}\right)$$

$$effSens_t \sim MVNormal\left(\left[\text{effSens}_{\mu,t}\right], \sigma_{effSens}\right)$$

$$\theta_t \sim MVNormal \sim \left(\left[\theta_{\mu,t}\right], \sigma_{\theta}\right)$$

S3. Individual parameter values at each timepoint were assumed to be drawn from multivariate normal distributions where $rewSens_{u,t}$, $effSens_{u,t}$ and $\vartheta_{u,t}$ are the group-level means for the parameter and timepoint, and $\sigma_{[t]}$ is the covariance between individual level parameters across timepoints. The prior correlation between

timepoints was uniform over [-1,1] using an LKJ(1) prior for all covariance matrices (Norbury et al., 2024); i=individual, t=game timepoint.

The parameter offsets $(rewSens_{i,t}, effSens_{i,t}, \widetilde{\theta_{i,t}})$ at a given time (main text equation 4 and 6) were derived using non-centred parameterization, which is recommended to separate the hierarchical parameters and lower-level parameters in the prior (33). If participant data was missing at a notification, the parameter was simply a function of the trait-apathy intercept $rewSens_{i,t} = (\beta_{traitR} \times apathy_i)$. For the model reported in Results, 14% of the choice and 9.7% of the self-report data was missing.

The recoverability of individual parameters was assessed by re-fitting the model to the model's predicted choices and self-reported state endorsements. In the recovery model, we set realistic, broad bounds on the group parameter variances (σ[0,10]) and on the individual parameter offsets for reward sensitivity, effort sensitivity, and state motivation [-10,10]. All parameters showed good to excellent recoverability (Pearson's *r* across all timepoints: reward sensitivity = 0.8, 95% CI [0.77, 0.82], effort sensitivity = 0.88, 95% CI [0.87, 0.9], state θ = 0.82, 95% CI [0.81, 0.83]).

Posterior predictive accuracy of the hierarchical generative model

We confirmed the model's posterior predictive accuracy as the match between choices and state endorsements generated stochastically from the posterior parameter estimates and task trials, compared with the observed data (means and SDs across participants reported). This was excellent for both choices (mean ± sem = 0.91 ± 0.002) and self-reported states endorsed by the model (0.83 ± 0.01). *Pseudo-*R^2 also indicated that the model was a good fit to the data compared with a chance model (*pseudo-*R^2 = 0.68). *Pseudo-*R^2 is the variance explained by the model compared to a chance model; 1-sum log $likelihood_{model}$ / sum (log(0.5)*t), where *t* is the number of trials and 0.5 is the likelihood of random behaviour in this task) (34) and here is similar to the previous study (Norbury et al., 2024). This indicates that the model was a good fit to the observed data.

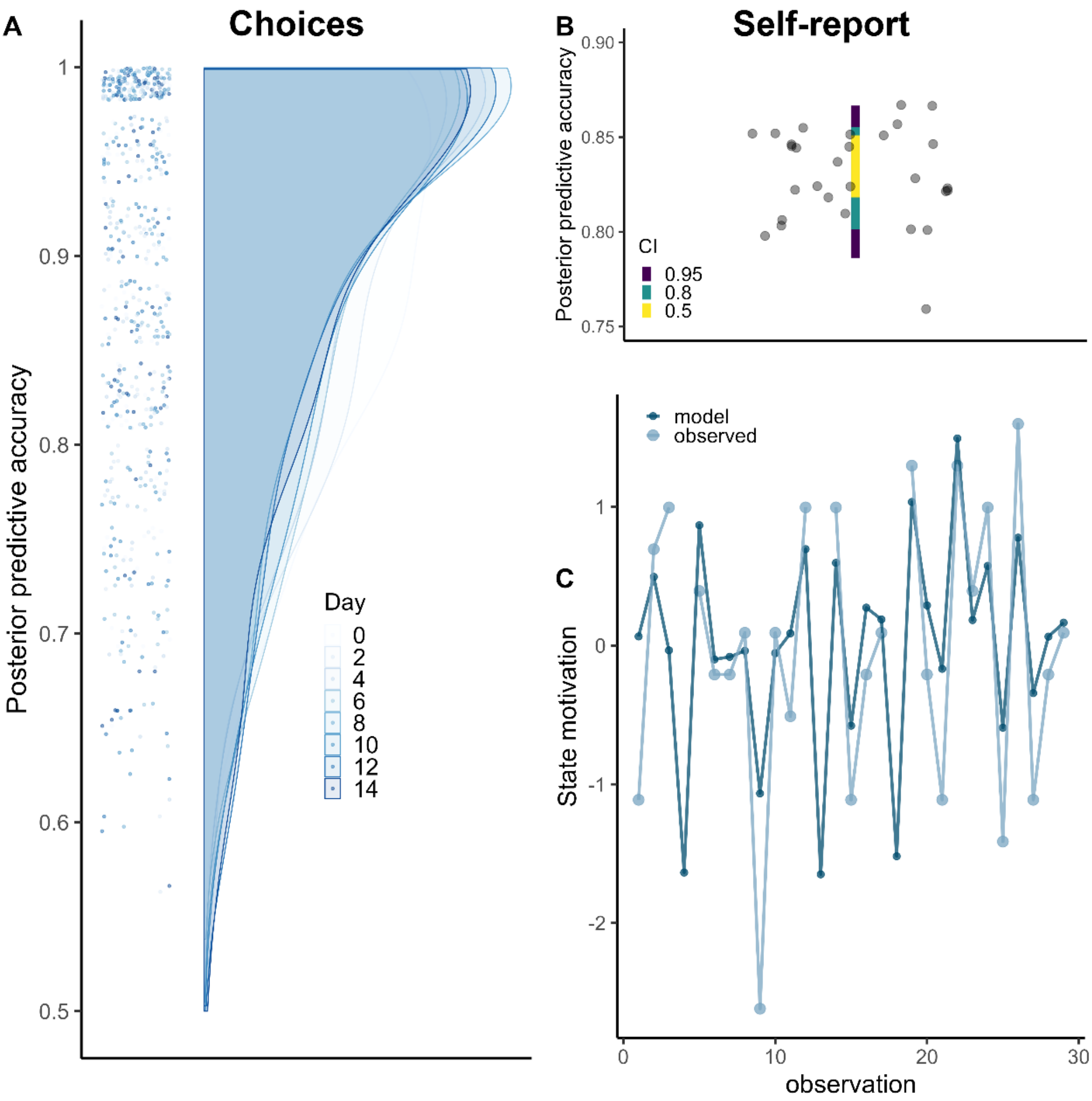

Supplementary Figure 11. Posterior predictive accuracy as a proportion of accurate model generated choices, where accuracy is defined as the model producing the same response as the participant. A: Mean posterior predictive accuracy of choices for each individual task that was played in the study. B: Mean posterior predictive accuracy across all participants for each state timepoint (total = 29). C: An example subject who exhibited the median correlation (Pearson's $r = 0.85$) between observed and model generated latent state motivation (θ) (y-axis) over time (x-axis).

Test re-test reliability of model parameters

Test re-test reliability of model parameter estimates was estimated within the generative hierarchical model following (1, 35, 36) which benefits from the shared covariance of related measurements and measurement error (precision) of individual estimates. Models were fit with a uniform prior on the correlation of individual values across timepoints [-1,1]. The

posterior test retest reliability correlation of individual parameter estimates across time-points is the test-retest reliability estimate. The mean re-test reliability of the subjective value parameters was good-excellent across all possible 2-game combinations (reward sensitivity = 0.93, 95% CI [0.91, 0.95], effort sensitivity = 0.76, 95% CI [0.73, 0.79]) The latent state θ was also moderately reliable (mean = 0.49, 95% CI [0.48, 0.5]) across all 29 timepoints over the 15 days. Since latent state θ was derived from items which asked participants to endorse how they felt "right now", lower test re-test reliability is what we would expect.

Trait-apathy and reward/effort sensitivity

Apathy is related to effort-based choices (21, 25, 37). In the model, we took an agnostic approach and allowed trait-apathy to independently influence both reward and effort sensitivity on a given day for each participant, with the prior on both effects centred on zero. This means that we took a data driven approach and allowed the model to find the β values which maximised the likelihood. In effect trait-apathy acted as an intercept on the parameter value (similar to a subject level intercept in the linear mixed effects model, with the value determined by the group effect and individual trait apathy score), which was also a linear function of the participant by time offset and state motivation (θ) and its effects (Main text, equation 5). The model-derived motivational state was strongly correlated with trait-apathy across people (according to 15-day mean, rho=0.63, p <0.001). To decorrelate the effect of trait-apathy from state motivation, the model-derived state was normalised within person which removed the correlation with trait apathy (rho=0.04, p = 0.65).

We use the term predict when describing the effects of trait-apathy because the score was obtained before the subsequent games (on day 0). We found that trait apathy did not significantly predict (90% CI for the group-level effects did not exclude zero) reward (β_{traitR} = -0.22 ± 0.23 (SD), 90% CI = [-0.59, 0.16], P(Direction) = 0.83); or effort sensitivity on any given day (effort sensitivity: β_{traitE} = 0.65 ± 0.49 (SD), 90% CI = [-0.15, 1.45], P(Direction) = 0.91). This is possibly because our sample did not include any clinically low trait motivation participants, and the intensive longitudinal design necessitated a high level of motivation to begin with. In summary, in this cohort the only significant relationship was person-centred state motivation on reward sensitivity (Supplementary Table 6).

Temporal effects of state motivation on reward sensitivity

The temporal effects of state on reward sensitivity were independently estimated post-hoc with a model which allowed the previous state (-1) and the next state in future (+1) to simultaneously influence reward sensitivity i.e.,

$$rewSens_{i,t} = (\beta_{traitR} \times apathy_i) + \widetilde{rewSens}_{\iota,t} + \left(\beta_{stateR-1} \times \theta_{i,t-1}\right) + \left(\beta_{stateR+1} \times \theta_{i,t+1}\right)$$

This model was fit only on games 2 through to 7, because games 1 and games 8 were completed at the first and last notification respectively (there were no previous or future states). To control for within and across day effects, the experimental design pseudorandomly counterbalanced the task order so that there were an even number of games in the morning and afternoon in this analysis (day2=AM, day4=PM, day6=PM, day8=AM, day10=PM, day12=AM). The effect of both the previous state ($\beta_{state-1}$) and the next state ($\beta_{state+1}$) were both assigned the empirical prior which we had identified for the effect of current state ($normal(0.19,0.08)$). Since the effect of state motivation on effort sensitivity was not significant, this was removed from the model such that

$$effSens_{i,t} = (\beta_{traitE} \times apathy_i) + \widetilde{effSens}_{\iota,t}$$

This temporal model was otherwise the same as the model previously reported (see Methods). The posterior parameter distributions for the effects of state-motivation in this model revealed that the previous state was coupled with reward sensitivity but the subsequent state in future was not (after controlling for the effect of the previous state, Results).

Given that the autocorrelation in state motivation was significantly greater than zero up until lag-2 (Results, Figure 2), we also tested whether the lag-2 state (state motivation from 2 timepoints in the past) also drove reward sensitivity, while controlling for the lag-1 association i.e.,

$$rewSens_{i,t} = (\beta_{traitR} \times apathy_i) + \widetilde{rewSens}_{\iota,t} + \left(\beta_{stateR-2} \times \theta_{i,t-1}\right) + \left(\beta_{stateR-1} \times \theta_{i,t+1}\right)$$

Again, we embedded the expectation that both effects were equal by assigning the same empirical prior (from the current state effect) to both t-1 and t-2 state effects:

$$\beta_{stateR-2} \sim normal(0.19,0.08)$$

$$\beta_{stateR-1} \sim normal(0.19,0.08)$$

Although the effect was predictably smaller, state motivation 2-timepoints in the past also significantly, positively predicted the next reward sensitivity ($\beta_{state\text{-}rewSens\text{-}2}$ = 0.08 ± 0.05 (SD), 90% CI = [0, 0.15], P(Direction) = 0.954).

Supplementary Tables with Posterior Parameter Estimates

Posterior parameter estimates: *reward & effort sensitivity*

Day	parameter	mean	se (mean)	sd	5%	95%	N_{eff}	$\hat{R}$
0	effort sensitivity	1.75	0.44	4.77	-2.68	6.10	4,763.22	1
2	effort sensitivity	1.82	0.49	5.35	-2.69	6.23	6,679.76	1
4	effort sensitivity	1.45	0.48	5.30	-3.11	5.89	7,467.00	1
6	effort sensitivity	1.32	0.43	4.67	-2.86	5.36	6,814.87	1
8	effort sensitivity	1.16	0.44	4.85	-2.98	5.19	7,450.36	1
10	effort sensitivity	1.06	0.39	4.22	-2.59	4.62	7,027.46	1
12	effort sensitivity	1.41	0.48	5.22	-3.19	5.93	6,894.65	1
14	effort sensitivity	0.87	0.38	4.17	-2.64	4.32	6,995.16	1
0	reward sensitivity	2.13	0.11	1.19	0.90	3.53	4,425.85	1
2	reward sensitivity	2.37	0.13	1.46	1.17	3.74	6,007.78	1
4	reward sensitivity	2.70	0.14	1.49	1.36	4.24	7,023.94	1
6	reward sensitivity	2.32	0.13	1.47	1.14	3.68	6,112.01	1
8	reward sensitivity	2.70	0.16	1.76	1.38	4.23	7,096.97	1
10	reward sensitivity	2.22	0.15	1.62	1.08	3.52	6,440.50	1
12	reward sensitivity	2.55	0.18	2.00	1.19	4.13	6,258.03	1
14	reward sensitivity	2.36	0.17	1.85	1.11	3.80	6,583.92	1

Supplementary Table 4. Summary for the posterior choice parameter values across the 15 days in the study. Mean=the posterior mean across all people on that day, se=standard error, sd = standard deviation, Neff=number of effective samples, $\hat{R}$ = convergence diagnostic where chains that have converged $\hat{R}$ is equal to 1.

Posterior parameter estimates: latent motivational state θ

t	mean	se (mean)	sd	5%	95%	N_{eff}	$\hat{R}$
1	0.34	0.06	0.65	-0.58	1.28	4,180.15	1
2	0.08	0.08	0.84	-0.93	1.13	6,140.56	1
3	0.21	0.06	0.69	-0.73	1.20	6,266.74	1
4	0.22	0.08	0.92	-0.78	1.24	6,081.56	1
5	0.18	0.08	0.84	-0.87	1.26	7,179.08	1
6	0.15	0.07	0.74	-0.83	1.16	6,654.82	1
7	0.02	0.06	0.67	-0.92	1.00	7,168.70	1
8	-0.16	0.07	0.78	-1.17	0.87	6,898.88	1
9	0.12	0.06	0.63	-0.83	1.10	6,855.23	1
10	-0.08	0.06	0.62	-1.00	0.87	6,600.11	1
11	0.18	0.08	0.87	-0.83	1.23	7,427.43	1
12	0.11	0.05	0.51	-0.81	1.06	7,030.12	1
13	0.34	0.07	0.80	-0.61	1.31	6,749.13	1
14	0.12	0.08	0.89	-0.95	1.23	7,571.17	1
15	0.02	0.08	0.90	-1.05	1.13	7,695.42	1
16	-0.09	0.06	0.68	-0.96	0.81	6,838.35	1
17	-0.18	0.05	0.54	-0.98	0.67	6,597.73	1
18	-0.09	0.06	0.61	-0.98	0.84	7,199.95	1
19	-0.17	0.06	0.67	-1.13	0.83	7,442.00	1
20	0.15	0.05	0.60	-0.84	1.18	8,049.53	1
21	-0.39	0.08	0.89	-1.32	0.57	6,850.65	1
22	0.03	0.07	0.78	-0.90	0.99	7,734.38	1
23	-0.02	0.07	0.72	-1.01	1.01	8,029.59	1
24	-0.27	0.08	0.90	-1.19	0.68	7,439.24	1
25	-0.30	0.06	0.71	-1.24	0.68	7,650.76	1
26	-0.30	0.05	0.55	-1.12	0.53	7,519.22	1
27	-0.03	0.07	0.78	-1.03	1.01	8,029.32	1
28	0.11	0.06	0.67	-0.85	1.11	7,782.86	1
29	-0.28	0.09	0.94	-1.20	0.66	6,684.10	1

Supplementary Table 5. Summary statistics from the posterior latent motivational state parameter (θ) values across the 29 state timepoints (t) in the study. Mean=the posterior mean across all people, se=standard error, sd = standard deviation,Neff=number of effective samples, $\hat{R}$ = convergence diagnostic where chains that have converged $\hat{R}$ is equal to 1.

Posterior parameter estimates for group level effects: β

parameter	level	domain	mean	sd	5%	95%	N_{eff}	*rhat*
β_{stateR}	state	reward	0.19	0.08	0.06	0.32	1,742.63	1
β_{stateE}	state	effort	0.29	0.32	-0.23	0.82	1,621.47	1
β_{traitR}	trait	reward	-0.22	0.23	-0.60	0.15	1,004.45	1
β_{traitE}	trait	effort	0.65	0.49	-0.16	1.44	2,371.81	1

Supplementary Table 6. Summary statistics for the posterior effects of interest (group level parameters) for the main effects of state and trait on each of reward sensitivity and effort sensitivity. Mean=the posterior mean across all people, se=standard error, sd = standard deviation,Neff=number of effective samples, $\hat{R}$ = convergence diagnostic where chains that have converged $\hat{R}$ is equal to 1.

Supplementary References

1. A. Norbury, T. U. Hauser, S. M. Fleming, R. J. Dolan, Q. J. M. Huys, Different components of cognitive-behavioral therapy affect specific cognitive mechanisms. *Sci. Adv.* **10**, eadk3222 (2024).

2. C. S. Kay, G. Saucier, The Comprehensive Infrequency/Frequency Item Repository (CIFR): An online database of items for detecting careless/insufficient-effort responders in survey data. *Personal. Individ. Differ.* **205**, 112073 (2023).

3. A. J. Barends, R. E. de Vries, Noncompliant responding: Comparing exclusion criteria in MTurk personality research to improve data quality. *Personal. Individ. Differ.* **143**, 84–89 (2019).

4. D. J. Hauser, N. Schwarz, Attentive Turkers: MTurk participants perform better on online attention checks than do subject pool participants. *Behav. Res. Methods* **48**, 400–407 (2016).

5. K. Thomas, S. Clifford, Validity and Mechanical Turk: An Assessment of Exclusion Methods and Interactive Experiments. *Comput. Hum. Behav.* **77** (2017).

6. S. Zorowitz, J. Solis, Y. Niv, D. Bennett, Inattentive responding can induce spurious associations between task behaviour and symptom measures. *Nat. Hum. Behav.* **7**, 1667–1681 (2023).

7. Y.-S. Ang, P. Lockwood, M. A. J. Apps, K. Muhammed, M. Husain, Distinct Subtypes of Apathy Revealed by the Apathy Motivation Index. *PLOS ONE* **12**, e0169938 (2017).

8. L. Daumas, *et al.*, Associations, overlaps and dissociations between apathy and fatigue. *Sci. Rep.* **12**, 7387 (2022).

9. C. Libedinsky, *et al.*, Sleep Deprivation Alters Effort Discounting but not Delay Discounting of Monetary Rewards. *Sleep* **36**, 899–904 (2013).

10. T. Müller, M. C. Klein-Flügge, S. G. Manohar, M. Husain, M. A. J. Apps, Neural and computational mechanisms of momentary fatigue and persistence in effort-based choice. *Nat. Commun.* **12**, 4593 (2021).

11. I. M. Berwian, J. G. Wenzel, K. E. Stephan, H. Walter, Q. J. M. Huys, Computational Mechanisms of Effort and Reward Decisions in Patients With Depression and Their Association With Relapse After Antidepressant Discontinuation. *JAMA Psychiatry* **77**, 513–522 (2020).

12. T. T.-J. Chong, *et al.*, Dopamine enhances willingness to exert effort for reward in Parkinson's disease. *Cortex* **69**, 40–46 (2015).

13. A. J. Culbreth, E. K. Moran, D. M. Barch, Effort-cost decision-making in psychosis and depression: could a similar behavioral deficit arise from disparate psychological and neural mechanisms? *Psychol. Med.* **48**, 889–904 (2018).

14. A. Dinno, *paran: Horn's Test of Principal Components/Factors* (2024).

15. L. W. Glorfeld, An Improvement on Horn's Parallel Analysis Methodology for Selecting the Correct Number of Factors to Retain. *Educ. Psychol. Meas.* **55**, 377–393 (1995).

16. J. L. Horn, A rationale and test for the number of factors in factor analysis. *Psychometrika* **30**, 179–185 (1965).

17. R. Schurr, D. Reznik, H. Hillman, R. Bhui, S. J. Gershman, Dynamic computational phenotyping of human cognition. *Nat. Hum. Behav.* 1–15 (2024). https://doi.org/10.1038/s41562-024-01814-x.

18. S. H. Lovibond, P. F. Lovibond, *Manual for the depression anxiety stress scales*, 2nd ed (Psychology Foundation of Australia, 1995).

19. S. N. Mullins-Sweatt, J. E. Jamerson, D. B. Samuel, D. R. Olson, T. A. Widiger, Psychometric Properties of an Abbreviated Instrument of the Five-Factor Model. *Assessment* **13**, 119–137 (2006).

20. R. P. Snaith, *et al.*, A Scale for the Assessment of Hedonic Tone the Snaith–Hamilton Pleasure Scale. *Br. J. Psychiatry* **167**, 99–103 (1995).

21. V. Bonnelle, *et al.*, Characterization of reward and effort mechanisms in apathy. *J. Physiol. Paris* **109**, 16–26 (2015).

22. H. Costello, M. Husain, J. P. Roiser, Apathy and Motivation: Biological Basis and Drug Treatment. *Annu. Rev. Pharmacol. Toxicol.* **64**, null (2024).

23. I. Groeneweg-Koolhoven, *et al.*, Apathy in early and late-life depression. *J. Affect. Disord.* **223**, 76–81 (2017).

24. S. R. C. Hewitt, J. Habicht, A. Bowler, P. L. Lockwood, T. U. Hauser, Probing apathy in children and adolescents with the Apathy Motivation Index–Child version. *Behav. Res. Methods* **56**, 3982–3994 (2024).

25. M. Husain, J. P. Roiser, Neuroscience of apathy and anhedonia: a transdiagnostic approach. *Nat. Rev. Neurosci.* **19**, 470–484 (2018).

26. P. Petitet, *et al.*, The relationship between apathy and impulsivity in large population samples. *Sci. Rep.* **11**, 4830 (2021).

27. M. Dubois, T. U. Hauser, Value-free random exploration is linked to impulsivity. *Nat. Commun.* **13**, 4542 (2022).

28. N. Haines, T. P. Beauchaine, Moving beyond Ordinary Factor Analysis in Studies of Personality and Personality Disorder: A Computational Modeling Perspective. *Psychopathology* **53**, 157–167 (2020).

29. A. Gelman, *et al.*, “Bayesian Workflow” (arXiv, 2020).

30. D. J. Schad, M. Betancourt, S. Vasishth, Toward a principled Bayesian workflow in cognitive science. *Psychol. Methods* **26**, 103–126 (2021).

31. Gabry, J., Češnovar, R., Johnson, A, *cmdstanr: R Interface to “CmdStan”* (2023).

32. A. Gelman, D. B. Rubin, Inference from Iterative Simulation Using Multiple Sequences. *Stat. Sci.* **7**, 457–472 (1992).

33. O. Papaspiliopoulos, G. O. Roberts, M. Sköld, A General Framework for the Parametrization of Hierarchical Models. *Stat. Sci.* **22**, 59–73 (2007).

34. N. D. Daw, “Trial-by-trial data analysis using computational models” in *Decision Making, Affect, and Learning: Attention and Performance XXIII*, (Oxford University Press, 2011).

35. N. Haines, H. Sullivan-Toole, T. Olino, From classical methods to generative models: Tackling the unreliability of neuroscientific measures in mental health research. *Biol. Psychiatry Cogn. Neurosci. Neuroimaging* **8**, 822–831 (2023).

36. J. N. Rouder, J. M. Haaf, A psychometrics of individual differences in experimental tasks. *Psychon. Bull. Rev.* **26**, 452–467 (2019).

37. M. Jurgelis, *et al.*, Heightened effort discounting is a common feature of both apathy and fatigue. *Sci. Rep.* **11**, 22283 (2021).
